# Genome-Wide Identification of Transcription Start Sites, Promoters and Transcription Factor Binding Sites in *E. coli*

**DOI:** 10.1371/journal.pone.0007526

**Published:** 2009-10-19

**Authors:** Alfredo Mendoza-Vargas, Leticia Olvera, Maricela Olvera, Ricardo Grande, Leticia Vega-Alvarado, Blanca Taboada, Verónica Jimenez-Jacinto, Heladia Salgado, Katy Juárez, Bruno Contreras-Moreira, Araceli M. Huerta, Julio Collado-Vides, Enrique Morett

**Affiliations:** 1 Departamento de Ingeniería Celular y Biocatálisis, Instituto de Biotecnología, Universidad Nacional Autónoma de México, Cuernavaca, Morelos, México; 2 Centro de Ciencias Aplicadas y Desarrollo Tecnológico, Universidad Nacional Autónoma de México, Cuernavaca, Morelos, México; 3 Programa de Genómica Computacional, Centro de Ciencias Genómicas, Universidad Nacional Autónoma de México, Cuernavaca, Morelos, México; 4 Unidad Universitaria de Secuenciación Masiva, Universidad Nacional Autónoma de México, Cuernavaca, Morelos, México; Baylor College of Medicine, United States of America

## Abstract

Despite almost 40 years of molecular genetics research in *Escherichia coli* a major fraction of its Transcription Start Sites (TSSs) are still unknown, limiting therefore our understanding of the regulatory circuits that control gene expression in this model organism. RegulonDB (http://regulondb.ccg.unam.mx/) is aimed at integrating the genetic regulatory network of *E. coli* K12 as an entirely bioinformatic project up till now. In this work, we extended its aims by generating experimental data at a genome scale on TSSs, promoters and regulatory regions. We implemented a modified 5′ RACE protocol and an unbiased High Throughput Pyrosequencing Strategy (HTPS) that allowed us to map more than 1700 TSSs with high precision. From this collection, about 230 corresponded to previously reported TSSs, which helped us to benchmark both our methodologies and the accuracy of the previous mapping experiments. The other *ca* 1500 TSSs mapped belong to about 1000 different genes, many of them with no assigned function. We identified promoter sequences and type of σ factors that control the expression of about 80% of these genes. As expected, the housekeeping σ^70^ was the most common type of promoter, followed by σ^38^. The majority of the putative TSSs were located between 20 to 40 nucleotides from the translational start site. Putative regulatory binding sites for transcription factors were detected upstream of many TSSs. For a few transcripts, riboswitches and small RNAs were found. Several genes also had additional TSSs within the coding region. Unexpectedly, the HTPS experiments revealed extensive antisense transcription, probably for regulatory functions. The new information in RegulonDB, now with more than 2400 experimentally determined TSSs, strengthens the accuracy of promoter prediction, operon structure, and regulatory networks and provides valuable new information that will facilitate the understanding from a global perspective the complex and intricate regulatory network that operates in *E. coli*.

## Introduction

Since the mid-1990s, the number of completely sequenced bacterial genomes has grown to more than 1350 and many more are in progress (http://www.ncbi.nlm.nih.gov/sutils/genom_table.cgi). These efforts have provided a vast amount of information regarding gene content, and thus the physiological traits of these organisms. Transcriptomic, proteomic and metabolomic experiments have greatly enriched our global understanding of the general metabolic potential of some of these bacterial species. Certainly, whole genome-expression profiles have made outstanding contributions to understand global gene expression patterns [1]–[5]. However, these data do not provide any molecular detail on the regulatory mechanisms that ultimately control or modulate gene expression, as promoter sequences, type of RNA polymerase (RNAP) σ factor and regulatory elements. Therefore, it is clear that significant contributions can be made by large-scale efforts aimed at identifying the major functional elements that typically control transcription initiation, such as promoters, DNA binding sites for transcription factors, and riboswitches. RegulonDB (http://regulondb.ccg.unam.mx/) is the primary reference database offering curated knowledge of the transcriptional regulatory network of *Escherichia coli* K12, currently the most used, electronically encoded, database of the genetic regulatory network of any free-living organism [6]. The main aim of this work was to greatly increase this knowledge by experimentally identifying as many TSSs, promoter sequences, and *cis*-acting DNA regulatory elements as possible.

The promoter element defines the DNA site directing the RNAP holoenzyme for transcription initiation, and it is a crucial element to understand gene expression in bacteria. Promoters differ at their consensus sequence depending on the interchangeable polymerase σ factor used, which provides DNA recognition specificity [7], [8]. The large number of experimentally determined promoter sequences for different σ factors in several organisms has allowed the use of prediction methods based on the generation of Position-Weight Matrices (PWM), which define conserved canonical motifs [9]–[11]. However, these methods produce a large number of false positive predictions with partial coverage, thereby limiting their usefulness [9]–[11]. Another level of complexity for reliable computational promoter prediction is the high density of promoter-like sequences in the extragenic regulatory regions of *E. coli*. We previously reported that putative promoters with scores higher than the experimentally determined promoters are detected in a great number of regulatory regions [12]. These patterns of unequal densities of σ^70^ promoter signals are a general trend in bacterial genomes, except for small intracellular parasites [13]. The above observations indicate that the prediction of functional promoters is very difficult. This intrinsic limitation of *in-silico* promoter prediction makes the experimental determination of the TSSs essential. Given the tightly constrained promoter position relative to the TSS (usually between 7 to 10 nucleotides) promoter identification is straightforward once the TSS has been experimentally determined.

The bacterial TSSs have been mainly identified through two experimental procedures: primer extension [14], and by S1 nuclease protection mapping assays [15]. These methods are labor intensive and differ in sensitivity. In *E. coli*, the genetically and physiologically most studied organism so far, more than 700 TSSs have been determined [16]. High throughput methodologies have been implemented for large scale TSSs mapping in *E. coli* using a tiled array approach [17]. A strong limitation of this methodology is that the TSSs were mapped to a window of about 30 nucleotides, which clearly is poor to make reliable promoter assignment. More recently, a step forward was achieved for reliable high throughput TSSs mapping in a genome-wide scale effort by determining 769 new TSSs of the bacteria *Caulobacter crescentus* with 5 nucleotide resolution [18]. Finally, Palsson and collaborators reported the identification of 1139 RNAP binding sites in *E. coli* using chromatin immunoprecipitation and microarrays (ChIP-chip). These experiments provided further evidence of active promoters but they were not designed to precisely map TSSs [19].

In this report we implemented a simple procedure based on a modification of the Rapid Amplification of 5′ complementary DNA ends (5′ RACE [20], [21]) named Directed Amplification of TSSs (DMTSS), and a high throughput pyrosequencing strategy (HTPS) with Roche's 454 GS20 instrument, to experimentally determine as many TSSs as possible in the *E. coli* K-12 genome. 317 putative TSSs were determined by DMTSS and about 1500 more by HTPS, constituting about two and a half times the TSSs mapped in more than 40 years of molecular genetic studies in this organism. Control experiments with genes for which the TSSs have been previously mapped showed that both methodologies are robust and accurate, and for many of them, additional TSSs not previously mapped were discovered, indicating that those genes are subject to a more complex genetic control than previously thought. Scrutiny of the promoter region of all the newly mapped TSSs helped us to identify their putative sequence and the most likely σ factor recognizing them. Remarkably, we found more than 2300 additional 5′ RNA ends by HTPS (with more than one replicate) within the coding regions, about 600 (26%) of them in antisense orientation, very likely with regulatory functions. In conclusion, our large-scale TSSs mapping effort adds substantial new information to the catalogue of experimentally determined promoters and DNA regulatory sites in *E. coli* that will encourage the experimental biologists to investigate the gene expression mechanisms underlying some of these genes, particularly for those with no biological function assigned. The methodologies described here can be applied to other less studied bacteria to gain functional insights into the transcriptional regulatory mechanisms that govern the spatio-temporal regulation of gene expression.

## Results and Discussion

### DMTSS Methodology for Genomic TSSs Mapping

**Figure 1:**
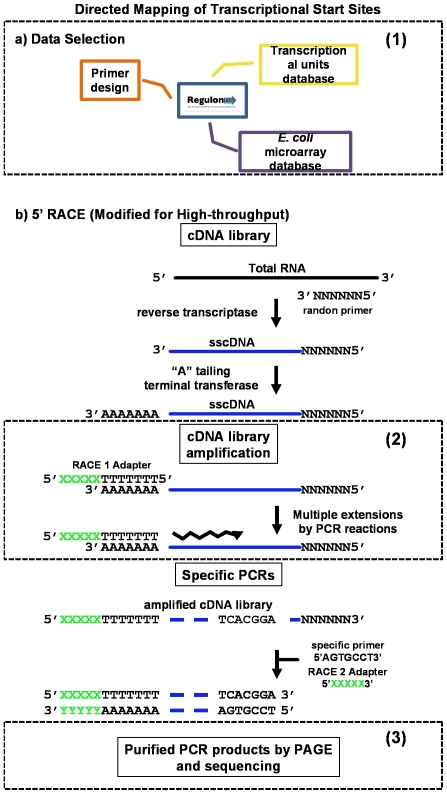
Directed Mapping of Transcription Start Sites (DMTSS). a) Data selection using different databases in regulonDB; b) Rapid Amplification of cDNA Ends modified protocol. The key points to enhance the efficiency of the DMTSS protocol for massive TSSs mapping were: 1) selection of highly expressed TUs under specific growth conditions, and rational oligonucleotide design; 2) lineal amplification of cDNA; 3) PAGE separation and purification of PCR products and sequencing.

The main objective of this study was to provide accurate identification of TSSs for a large number of *E. coli* K-12 transcriptional units (TUs). The precise mapping of the TSSs is critical to unambiguously identify promoters and gene expression regulatory sequences. We implemented two methodologies to map TSSs, HTPS (see below), and DMTSS. For the latter, total mRNA is randomly amplified, and the resulting cDNA is labeled at the 3′ end by incorporating a homopolynucleotide. This pool of cDNA can be used in independent experiments to map hundreds of TSSs using gene-specific oligonucleotides as primers in a PCR reaction (Figure 1B). As with the great majority of the reports in the literature, the TSS determination here is indirect, since we did not determined 5′ triphosphate ends.

### Robustness of the DMTSS Methodology for Genomic TSSs Mapping

**Figure 2:**
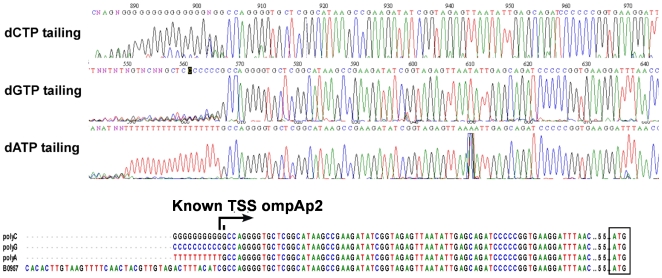
Analysis of different 3′ end polynucleotide incorporation efficiency. A) Electropherograms show the incorporation of dCTP, dGTP, and dATP at the 3′ end of the cDNA for precise map the TSS of the *ompA* gene (ompAp2*) [51]. dATP was the one that produced the most homogeneous tail. B) Sequence comparison shows the 5′ end of the different tailing reactions.

**Figure 3:**
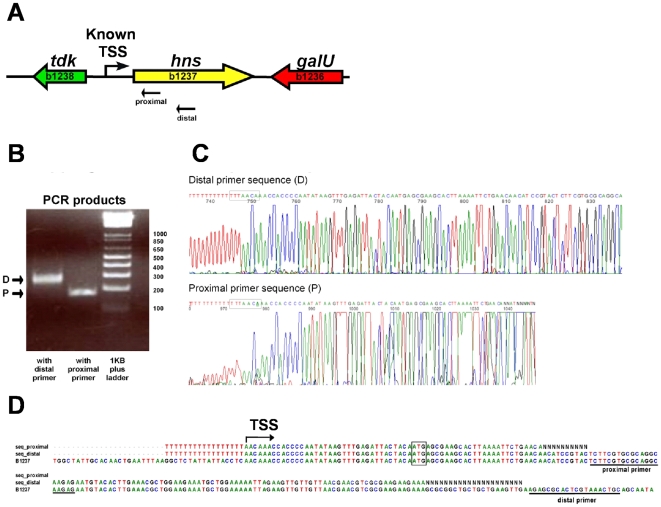
Mapping the TSS of the *hns* gene. A) Proximal and distal oligonucleotides were designed to prime 38 and 155 nucleotides downstream of the ATG, respectively. B) The PCR products generated with each oligonucleotide primers were separated by PAGE and purified from the gel. C) Nucleotide sequence of each PCR band after excision from the gel. The nucleotide immediately before the polynucleotide tail corresponds to the TSS. D) Comparison of the nucleotide sequences obtained with the TSS previously reported [22].

**Figure 4:**
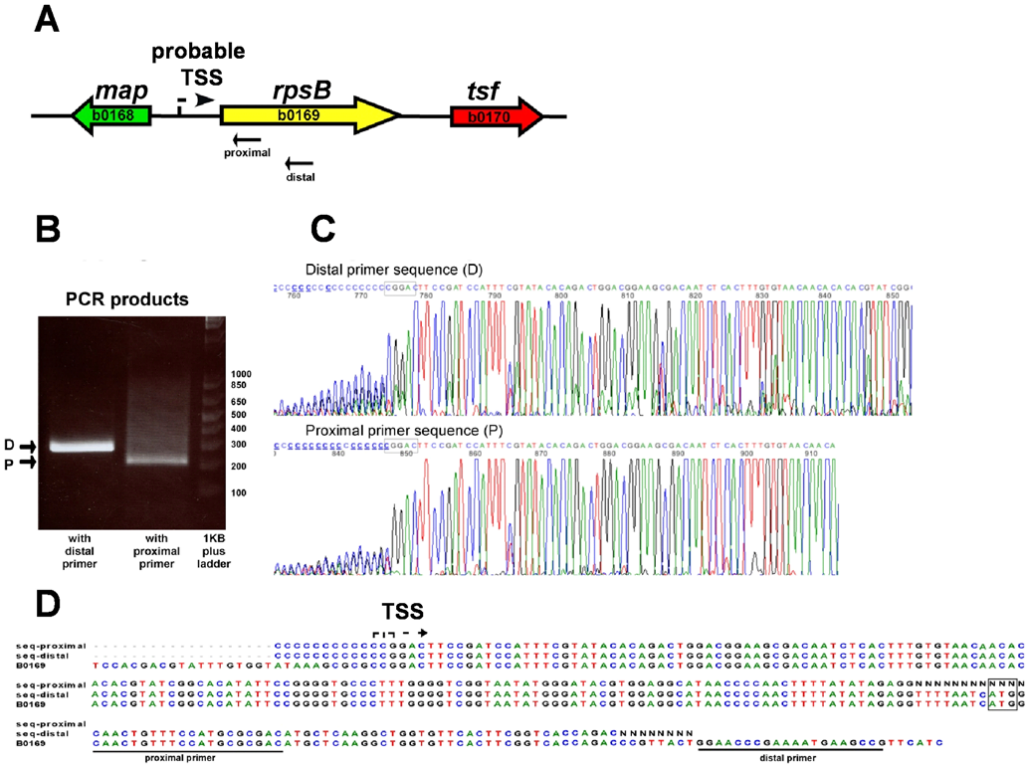
Determination of the unknown TSSs for *rpsB* gene. A) Proximal and distal oligonucleotides were design to prime 4 and 67 nucleotides downstream of the ATG, respectively. B) The PCR products generated with the oligonucleotide primers were separated by PAGE and purified from the gel. C) Nucleotide sequence of each PCR band after excision from the gel. The 3′ end the nucleotide immediately before the polynucleotide tail is the TSS. D) Comparison of the nucleotide sequences obtained with upstream region of *rpsB*.

First, we evaluated which nucleotide whose homopolymer produced the most defined 3′ end sequence when added by the terminal transferase. Each of the four nucleotides was used for tailing; as seen in Figure 2, labeling the 3′ end of the cDNA with polyA produced the clearest sequencing electropherogram, thus, although we detected this effect only for a single gene, we choose adenine for tailing. Second, to test the robustness of the methodology, we mapped TSSs for TUs that have already been determined by other groups. Eighteen genes whose TSSs have been previously reported (Table 1, genes labeled with a) that differed in their expression levels were selected, and a set of two gene-specific oligonucleotides that prime about 100 nucleotides apart, were designed for each gene with the GPA program (Huerta, AM. *et al*, “Genome Primer Analysis: A Web-Based Tool to Design gene-specific oligonucleotide primers for mapping 5′-ends of Bacterial RNA.”, manuscript in preparation; see Materials and Methods). Thus, two different extension products for each TU would be generated, one about 100 nucleotides longer than the other. Sequencing the 3′ end of each extension product should produce the same sequence, regardless of the length of the extension product. As an example, Figure 3A shows the genome region of the *hns* gene, and a schematic representation of the two oligonucleotides used as primers to map its TSSs. Figure 3B shows the extension products for each oligonucleotide; the difference in size of these products corresponded roughly to the distance of the primers in *hns* gene. The bands were purified from the gel and sequenced: electropherograms showed that both extension products generated the same 3′ end (Figure 3C), corresponding to the complementary 5′ end of the mRNA and, therefore, to the TSS. Comparison of the two sequences with the *E. coli* genome (Figure 3D) pointed exactly to the *hns* TSS previously mapped [22], [23]. For the rest of the control genes, two extension products differing roughly by 100 bp in length were also observed. The longest products were sequenced and the 3′ end of each of them corresponded precisely to the TSS previously mapped, except for *acs* and *ompA*, which differed by one nucleotide with the TSS reported in the literature (Table 1B). Several replicates (up to eight times for the *rpsP* gene) were carried out for these control genes, and always the same TSSs were detected (Figure S1). For a small number of genes with unknown TSSs we also used the double oligonucleotide strategy. Figure 4 shows the result of the TSS mapping for the *rpsB* gene. Both extension products and their corresponding sequences pointed to the same TSS.

**Table 1:** Results obtained with DMTSS compared with previously reports of TSSs. In the cases with a * mark, additional TSSs were identified.

**A) perfect match**							
**Bnumber (name)**	**Antecedent**	**Promoter**	**Known TSS**	**DMTSS**	**Diffrence**	**Reference**	
B0023(rpsT)	a	p1	−132	−132	---	Mackie GA., 1986	
B0118(acnB)*	a	p1	−96	−96	---	Cunningham L., 1997	
B0710(ybgL)	c	p2	−29	−29	---	Gifford CM., 2000	
B0733(cydA)	a	p2	−174	−174	---	Cotter PA., 1997	
B0759(galE)	b	p1	−26	−26	---	Colland F., 1999; Tanaka K., 1995	
B0871(poxB)	a	p1	−27	−27	---	Chang YY., 1994; Wise A., 1996	
B0929(ompF)	a	p1	−110	−110	---	Batchelor E,2005.	
B1015(putP)	c	p1	−137	−137	---	Nakao T., 1987	
B1015(putP)	c	p5	−13	−13	---	Nakao T., 1989	
B1237 (hns)	a	p1	−36	−36	---	La Teana A., 1989	
B1276(acnA)*	a	p2	−50	−50	---	Cunningham L., 1997	
B1415(aldA)	a	p1	−42	−42	---	Limon A., 1997; Pellicer MT., 1999	
B1641(slyB)	c	p1	−99	−99	---	Minagawa S., 2003	
B1661(cfa)	c	p1	−212	−212	---	Wang AY., 1994	
B1677(lpp)	b	p1	−38	−38	---	Nakamura K., 1979	
B1761(gdhA)*	b	p1	−63	−63	---	Riba L., 1988	
B1779(gapA)	b	p1	−36	−36	---	Charpentier B., 1994; 1998; Thouvenot B., 2004	
B2096(gatY)	a	p1	−30	−30	---	Nobelmann B., 1996	
B2215(ompC)*	b	p1	−81	−81	---	Huang L., 1990	
B2240(glpT)	b	p1	−77	−77	---	Larson TJ., 1992; Yang B., 1997	
B2414(cysK)*	a	p1	−32	−32	---	Byrne CR., 1988	
B2609(rpsP)	a	p1	−34	−34	---	Bystrom AS., 1989	
B2997(hybO)*	c	p1	−102	−102	---	Richard DJ., 1999	
B3298(rpsM)*	a	p1	−94	−94	---	Post LE., 1980	
B3365(nirB)	b	p1	−24	−24	---	Harborne NR., 1992	
B3426(glpD)	b	p1	−42	−42	---	Yang B., 1996; Ye SZ., 1988	
B3495(uspA)	b	p1	−128	−128	---	Nystrom T., 1992; Nystrom T., 1994	
B3528(dctA)	a	p1	−51	−51	---	Davies SJ., 1999; Wang YP., 1998	
B3707(tnaC)	b	p1	−24	−24	---	Deeley MC., 1982	
B3916(pfkA)*	a	p1	−78	−78	---	Hellinga HW., 1985; Crooke H., 1995	
B3961(oxyR)	b	p1	−33	−33	---	Tartaglia LA., 1989	
B4000(hupA)	a	p1	−105	−105	---	Claret L., 1996; Kohno K., 1990	
B4025(pgi)	a	p1	−36	−36	---	Froman BE., 1989	
B4034(malE)	b	p1	−45	−45	---	Bedouelle H., 1983; Richet E., 1996	
B4177(purA)	b	p1	−23	−23	---	Makaroff CA., 1985	
B4233(mpl)	c	p2	−25	−25	---	Talukder AA., 1996	
**B) imperfect match (+/− 1–2)**							
**Bnumber (name)**	**Antecedent**	**Promoter**	**Known TSS**	**DMTSS**	**Diffrence**	**Reference**	**Method**
B0436(tig)*	c	p1	−139	−140	−1	Aldea M., 1989	nuclease mapping
B0865(ybjP)*	c	p1	−56	−55	1	Lacour S., 2004	primer extension
B0910(cmk)	c	p1	−36	−38	−2	Pedersen S., 1984	nuclease mapping
B0957(ompA)	a	p2	−134	−133	1	Cole ST., 1982	nuclease mapping
B1015(putP)	c	p4	−95	−94	1	Nakao T., 1987	nuclease mapping
B1594(dgsA)	b	p2	−27	−26	1	Decker K., 1998	nuclease mapping
B1661(cfa)	b	p2	−34	−33	1	Wang AY., 1994	primer extension
B2313(cvpA)	b	p1	−37	−36	1	Makaroff CA., 1985; Nonet ML., 1987	nuclease mapping
B4069(acs)	a	p2	−20	−19	1	Beatty CM., 2003	nuclease mapping
B4149(blc)	c	p1	−23	−25	−2	Bishop RE., 1995	primer extension
**C) ambiguity by tailing**							
**Bnumber (name)**	**Antecedent**	**Promoter**	**Known TSS**	**DMTSS**	**Diffrence**	**Reference**	**Ambiguity**
B0166(dapD)	b	p1	−31	−30	1	Richaud C., 1984	tg
B0439(lon)	c	p1	−73	−76	−3	Chin DT., 1988	ta
B1101(ptsG)	c	p1	−103	−102	1	Plumbridge J., 1998	ta
B1421(trg)	c	p1	−33	−32	1	Park K., 2001	tc
B1493(gadB)	c	p1	−27	−28	−1	Castanie-Cornet MP., 2001	tg
B1866(aspS)*	c	p1	−95	−94	1	Eriani G., 1990	ttttttc
B3930(menA)*	c	p1	−57	−53	4	Suvarna K., 1998	ttttc
B4123(dcuB)	b	p1	−20	−19	1	Golby P., 1998	tta

a) original control; b) fortuitously mapped; and c) later annotated.

**Figure 5:**
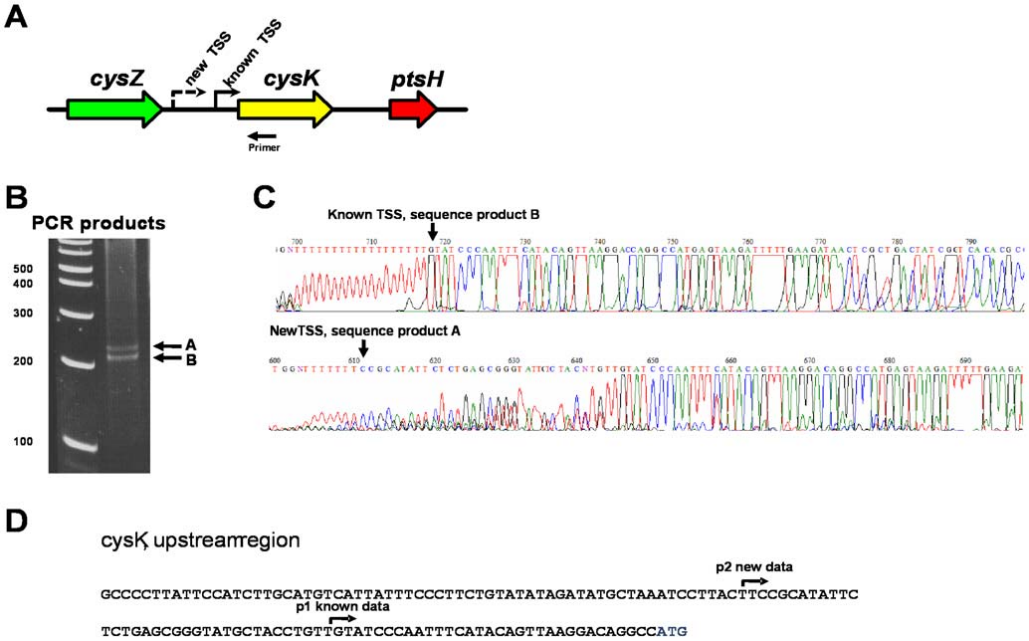
Mapping the TSSs of the *cysK* gene. A) The oligonucleotide primer was designed to prime 97 nucleotides downstream of the ATG. B) The A and B PCR products generated with the oligonucleotide primer were separated by PAGE and purified from the gel. C) Nucleotide sequence of each PCR band. The 3′ end the nucleotide immediately before the polynucleotide tail is the TSS. D) Comparison of the nucleotide sequences obtained with the upstream region. The previously reported TSS [52] was located 34 nucleotides upstream from the ATG, while the new TSS was located at 67 nucleotides downstream.

The above results indicate that the DMTSS methodology is accurate, robust and highly reproducible. It also helped us to determine the ideal length of the extension products. Interestingly, in one third of the control genes (*ompF*, *cysK*, *rpsM*, *pfkA*, *acnB* and *acnA*) we detected an additional TSSs not previously reported (detected as additional bands of different size using the same gene-specific oligonucleotide, and confirmed by nucleotide sequence). Putative promoters with significant scores (see below) were detected in front of many of these new TSSs (Table S1), implying that they are indeed RNAP transcription initiation events. Figure 5 shows the TSS mapping for *cysK*. The new data indicates that these genes are differentially expressed and subjected to a more complex genetic regulation than previously thought. This observation clearly exemplifies the advantage of the genome-wide DMTSS approach to provide insights into the regulatory elements controlling gene expression.

### TSSs Determination of Target Transcriptional Units

According to RegulonDB (version 6), *E. coli* K-12 contains 3133 TU, either as a single gene or as polycistronic operons [16]. 791 of them have experimentally determined and annotated TSSs. We selected a set of highly expressed TUs, according to microarray expression data, that lack experimentally determined TSSs (see Materials and Methods). The DMTSS experiments were performed following a descending expression order, assuming highly expressed genes would most likely produce unambiguous 5′ ends, and we stopped the experimental search when the success rate was low.

**Figure 6:**
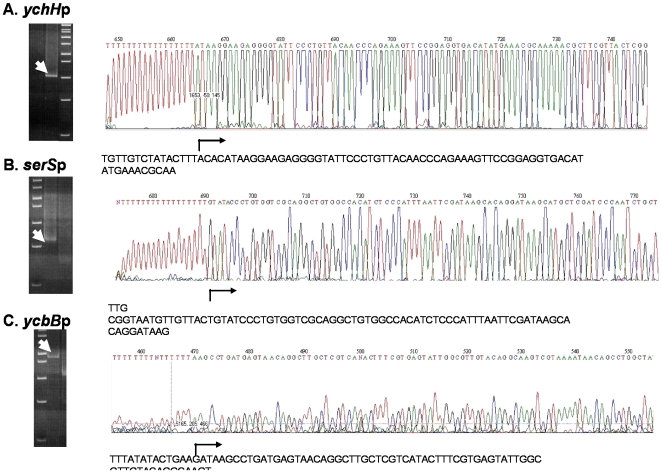
TSSs mapping for three genes with no previously determined 5′ end, as examples of the 317 TSSs mapped in this work. The TSSs for *ychH* (A), *serS* (B), and *ycbB* (C) genes, which code for a predicted inner membrane protein, a seryl-tRNA synthetase, and a predicted carboxypeptidase, respectively, were determined by DMTSS. The unique PCR fragments obtained by PCR for each gene were sequenced. The positions of the TSSs are indicated by arrows.

In total, 623 TUs were analyzed: gene-specific oligonucleotide primers were designed for the first gene of each of these TUs (Table S2), and we proceeded to independently generate extension product(s) for every one of them. In general, we obtained from one to few extension products per oligonucleotide. For 317 (about 51%) we were able to map with high confidence the 5′ end(s); for the rest, the poor quality or complete absence of specific PCR amplification products and/or sequencing reactions, precluded us from unambiguously obtaining the TSSs. This could be due to instability and/or low abundance of some of these mRNA transcripts, and/or that the target genes are not the first genes in their respective TU. Thus, in this report we included only the TSSs for which very clear 5′ ends were detected (Table S1 and Figure S2). The complete experimental results for each TSS determination is in: http://www.ccg.unam.mx/Computational_Genomics/SupMaterial/TSS/index.html. Figure 6 shows three different mapping experiments for TUs that did not have TSSs reported. As can be seen, well-defined 5′ ends were detected in all of them.

Of the 317 TSSs mapped by DMTSS, 263 did not have previous experimental evidence, while 54 had been reported and served us as controls (Table 1). As discussed above 18 of them were initially used to evaluate the methodology, 17 more were fortuitously mapped due to partial sequence complementary (from eight to eleven identical positions at the 3′ end) of some oligonucleotide primers designed for other genes. As an example, Figure S3 shows the extreme case of an oligonucleotide designed for *ybbA* with partial complementarity with the *gdhA* and *sstT* genes. This limited base pair complementary was sufficient to generate extension products and to solve the TSSs for these two genes (revealing two TSSs for *sstT* gene). The other 19 TSSs were annotated in RegulonDB after we started this work (this database is continuously updated). Detailed examination of these controls confirmed that the DMTSS methodology is very robust. 67% coincided precisely with the previously mapped TSSs; 29% had a small difference in the position of the TSSs of up to two nucleotides; and the remaining 4% had a difference of up to 3 nucleotides, but there was some uncertainty of where is the 5′ end due to “ambiguity by tailing”. This ambiguity arises when the last nucleotide of the sequence is the same as the polynucleotide tail that is used to label the 3′end of the cDNA library, such that it is not possible to determine where exactly the tailing begins. This problem is easy to overcome if a different nucleotide is used to generate the 3′ tail of the cDNA. Figure S4 shows a typical case of “ambiguity by tailing” and how the TSS was precisely determined.

Since our strategy did not select for any particular class of genes other than the most highly expressed ones, we ended up mapping the TSSs of 130 genes for which function is not known (predicted, conserved, putative, hypothetical or unknown; Table S3). Very few of this class of genes have their TSSs mapped in *E. coli*. Our unbiased genomic approach identified the TSSs, promoters, and in some cases even regulatory binding sites (see below) for a large number of genes with no precise function assigned. We expect that this new data will contribute in some cases to narrow down the function of these genes.

### Operons With More Than One TSS

**Figure 7:**
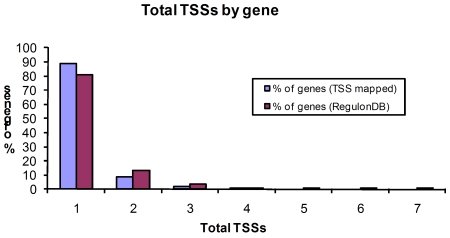
Number of TSSs per gene mapped. Comparison of the TSSs obtained in this work with the ones in RegulonDB. Both data sets are very similar, indicating no bias in the genes selected in this work.

**Figure 8:**
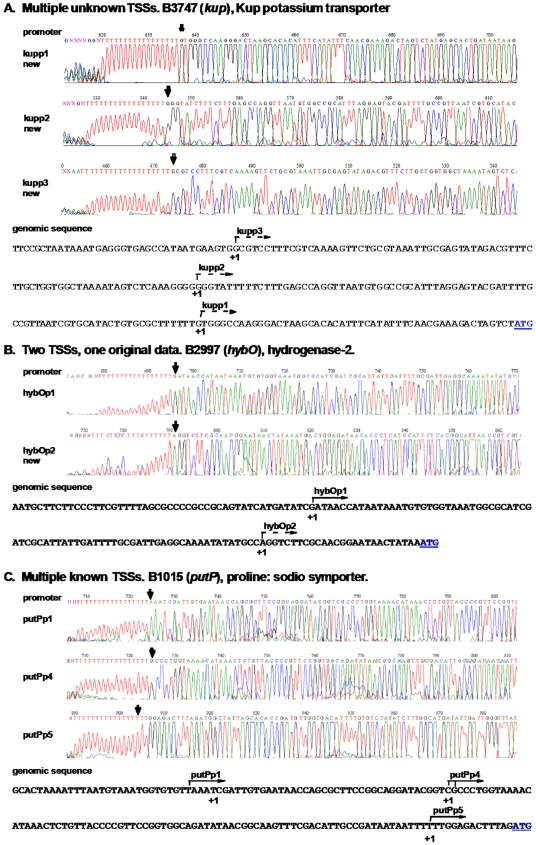
DMTSS results. A) Multiple new TSSs were obtained for the *kup* gene, 57, 135, and 213 nucleotides upstream of the ATG. B) A new TSS for *hybO* was identified 26 nucleotides upstream of the ATG, plus the previously reported one at 102 nucleotides upstream of the ATG. C) For *putP* three TSSs out of five reported were mapped 17, 94, and 138 nucleotides upstream of the ATG.

The 317 TSSs mapped by DMTSS correspond to 269 genes, 48 of these genes have more than one TSS. Figure 7 shows the percentage and number of TSSs per gene. There were up to four TSSs for a single gene. We compared these data to the number of TSSs per gene reported in the literature and, as shown in Figure 7, the results were very similar, showing that we detected the majority of the TSSs per gene. As an example of the newly determined TSSs, Figure 8A describes the mapping of the 5′ end products for the potassium transporter gene *kup*. There is a single known TSS for the *hybO* gene (Figure 8B) [24] yet we found a second one 75 nucleotides downstream. Finally, (Figure 8C) five TSSs have been reported for the *putP* gene [25]. We were able to map three of them: two that matched to the reported ones, while for the other there is one nucleotide difference. It should be pointed out that the TSSs found by us and by others represent only those that are expressed under the conditions tested. It is very likely that more TSSs could be found if experiments were carried out in several different growing conditions, as distinct promoters controlling the same operon are used differentially, depending on the environmental or growth conditions of the cell, as seen for *infA* gene expression in response to cold shock [26]

### TSSs Within the Coding Region Detected by DMTSS

Our dataset contains 21 cases where the 5′ end of the transcript lies within the coding region of their respective genes, including *gspA* for which two TSSs within the coding region were detected. Since a full length protein cannot be produced if the 5′ end of the mRNA is missing, it is possible that these results could be the due to misprediction of the protein start codon annotation, specific mRNA degradation, or *bona fide* secondary TSSs whose functionality is unknown. To discern if the apparent discrepancy comes from an incorrect genome annotation, *ie* the predicted initiation codon is not the used by the cell, we carried out BLASTP searches of the NH_2_ terminal fragments of each coded protein against the NCBI Proteobacteria database. These analyses indicated that the ORF annotation was incorrect for the *ygbI* and *ybjX* genes. YgbI orthologs are 10 amino acids (30 nucleotides) shorter than the *E. coli* putative protein; thus, using this as the genuine initiation codon then the TSS determined by us maps 21 nucleotides upstream of it. For YbjX, all proteobacterial orthologs, including the closely related *Salmonella* species, the protein initiates at a methionine that is at position 13 of the putative *E. coli* protein. Therefore, it is also very likely that in *E. coli*, the YbjX protein initiates further downstream and therefore the TSS is now at position −18. These results indicate that TSS mapping can also help in some cases to properly annotate ORFs. On the other hand, if the rest of the TSSs are *bona fide* transcription initiation events, they must come from a promoter. Therefore, we analyzed the region upstream of each TSSs and for 16 of them clear σ^70^ or σ^38^ −10 recognition elements with high scores from two different prediction methods (see below) were detected at the right position upstream of some 5′ ends described in this section (Table 2). For example, the *hdeD* gene that encodes an acid-resistance membrane protein, presents a consensus σ^38^ −10 recognition element, while *ygbI* has a σ^70^ −10 recognition element, thus very likely, they are genuine promoters. It seems that the 5′ ends of these 16 genes come from RNAP initiation events whose functionality is unknown. Interestingly, in half of these genes there are other(s) TSSs upstream of the ATG (Table 2), so the protein can be produced in full length. For the remaining five cases, we cannot rule out that the observed 5′ end is the result of specific mRNA degradation, and for three of them other TSSs upstream of the ATG were detected. Several hundred TSSs were also detected by HTPS (see below).

**Table 2:** Prediction of putative σ^70^ and σ^38^ −10 elements at the 5′ ends detected within coding regions.

Promoter	ATG dist.	−10 element	PATSER score	σ factor	other 5′ end	ATG dist.
*ycaO*p	+167	ttgcCGTATTATcgtg	3.64	σ70	…	…
*yi2_3*p	+17	aagtGATAGTCTtaat	3.21	σ70	…	…
*yjfO*p	+5	aggcACTACACTATggtt	5.16	σ 38	…	…
*hdeD*p	+16	tggtTCTATGTTATatat	4.03	σ 38	[51]	−35
*degQ*p	+46	gtgcATTAGCGTtaag	2.81	σ 70	This work	−90
*ygbI*p	+9	atagCGTAGAATgtca	3.56	σ 70	…	…
*yceA*p	+121	Non predictions	…	…	[50]	−35
*fabI*p	+161	Non predictions	…	…	This work	−82
*trmJ*p	+53	Non predictions	…	…	…	…
*ygaZ*p	+72	Non predictions	…	…	…	…
*gspA*p6	+65	Non predictions	…	…	[52]	−97,−112, −121,−236
*gspA*p5	+19	Non predictions	…	…	[52]	−97,−112, −121,−236
*fre*p	+131	Non predictions	…	…	…	…
*yeg*H	+26	Non predictions	…	…	…	…
*ych*Pp	+24	Non predictions	…	…	…	…
*ybjX*p	+18	Non predictions	…	…	…	…
*acnB*p3	+47	Non predictions	…	…	[53]	−97
*dapF*p	+117	Non predictions	…	…	…	…
*mobA*p	+43	Non predictions	…	…	[54]	−31,−122
*tktA*p	+92	Non predictions	…	…	This work	−76
*rnep*	+44	Non predictions	…	…	[55]	…

The PATSER program [39], was used to search for conserved −10 elements for both sigma factors upstream of the 5′ ends located within the putative coding region. The TSSs previously reported are indicated.

We would like to point out that it is not surprising to find fortuitous initiation events. The information content of the minimum promoter is low, such that in the whole genome of *E. coli* there must be several sequences that by chance resemble promoters that could be mistaken as such by the transcriptional machinery. If this is indeed the case, it implies that the cell is robust enough to tolerate fortuitous initiations events at non-genuine promoters. In addition, if these promoter-like sequences were affecting cell fitness they would be selected against. Recently, it has been documented that in an assumed homogeneous bacterial culture there are many local differences due in part to different stochastic events [27]. At least some of these could be the result of fortuitous initiation events that affect to a certain level the behavior of the cell.

### TSSs for Small RNAs

In addition to the TSSs mapped for mRNA, three TSSs that corresponded to small RNAs were determined. Those are *csrB*, *tff* and *sroG* (Figure S5). The first has been implicated in the accumulation of glycogen in stationary growth phase [28]. The function of the second and the third small RNAs, which are in front of the *rpsB* and *ribB* genes is not known. In both cases, the small RNAs form part of an operon with the adjoining genes. It will be very interesting to elucidate the processing mechanism that generates these small RNAs out of the large primary transcripts.

### Promoter Type Determination

Transcription initiation occurs when the RNAP, associated with one of the seven σ factors present in *E. coli*, recognizes and productively bind to specific promoter DNA sequences. Each σ factor recognizes promoter motifs that differ in their consensus DNA sequence. The majority of the genes that are expressed during exponential growth are transcribed by the RNAP with the “housekeeping” σ^70^ factor. The other six “alternative” σ factors have specific roles in stress survival and adaptation to environmental conditions, such as growth transitions and morphological changes [29], [30]. Unambiguous determination of which form of RNAP holoenzyme is transcribing a gene is not straightforward. The canonical model for the σ^70^-DNA promoter sequence is a −35 hexamer, separated by 15 to 21 nucleotides from the −10 hexamer. The consensus sequence reported for the −10 and the −35 elements are TATAAT and TTGACA, respectively [31]–[35]. Additionally, it is well known that other elements can modify the canonical model of the σ^70^ promoters, for instance the extended −10 region (TG), that is located 1 nucleotide upstream of the −10 element, and the UP element, usually positioned 4 nucleotides upstream of the −35 promoter region [36], [37]. Promoters that contain the extended −10 region normally have a −35 element less well conserved or absent, hence making these sequences dispensable for promoter function. Unlike the −35 element which appears to be more heterogeneous in sequence, the −10 element plays an essential role in promoter binding by stabilizing the initial interactions between the RNAP holoenzyme and the DNA promoter sequence, and consequently facilitating the subsequent isomerization of the promoter to an open form, exposing the DNA template strand [38]. The different particular combinations of the elements mentioned above contribute to alter or vary the basal promoter strength, and generate an enormous functional diversity among the σ^70^ promoters. On average, σ^70^-depended promoters preserve eight of the 12 canonical nucleotides of the −35 and −10 hexamers [34], [38], however around 10% of promoters match the consensus in only about half the nucleotides and yet they serve as sites for σ binding.

We anticipated that the majority of the TSSs mapped here are the result of promoters recognized by E-σ^70^, followed by those depended on σ^38^. To annotate the most likely promoters and the σ factors recognizing them we used the Position-Weight Matrices (PWM), generated by Huerta and Collado-Vides [12] for σ^70^ and for σ^38^ we generated a new PWM using the WCONSENSUS program. Each PWM was scanned with the PATSER program, in order to search for patterns in regions upstream of the TSSs obtained by DMTSS [39] (See Materials and Methods). For the 317 TSSs obtained by DMTSS, we identified 204 σ^70^ and 14 σ^38^ promoters with high levels of confidence (Table 3). For the rest, it is likely that they either are below the threshold used for detection, or that they are transcribed by other σ factor not analyzed here.

**Table 3:** Promoters identified by DMTSS.

DMTSS approach	Total	Other Evidence	New Data
−10 element σ^70^	140	22	118
−35 element σ^70^	35	6	29
−10/−35 elements σ^70^	29	7	22
−10 element σ^38^	14	3	11
Total	218	38	180

Interestingly, the distribution of scores between genes with known function and those with no clear function assigned is similar, adding further evidence that these putative ORFs are indeed genes that are transcribed by *bona fide* RNAP initiation events.

In the accompanying article (Olvera, L. *et al*), we investigated the TSSs and the promoter structure of all the genes involved in the glycolytic pathway in *E. coli* wild type and in some mutant derivatives lacking the phosphotransferase system. The great majority of these genes have overlapping promoters for σ^70^ and σ^38^, and thus can be transcribed by both RNAP holoenzymes depending on the growth or stress conditions of the cell.

### Genomic TSSs Determination by Pyrosequencing

The results shown above demonstrate that the DMTSS is a robust and sensitive method that can be used to map hundreds of TSSs. However, as the traditional methods for TSS mapping, it is based on the individual search of 5′ mRNA ends using gene-specific oligonucleotides. In recent years three different high throughput DNA sequencing platforms have been developed [40]. We considered that pyrosequencing by Roche's 454 GS technology [41] could be used to detect cDNAs corresponding to the 5′ ends of mRNAs expressed in a particular growth condition without any bias. To that end, we modified Roche's amplicon DNA sequencing protocol to sequence cDNAs up to their 3′ end, as described in Materials and Methods. The basis of this strategy is the use of an oligonucleotide similar to Primer B of Roche's protocol but with an additional random hexamer at the 3′ end. Instead of ligating oligonucleotide B to one end of the fragmented DNA molecules, this modified oligonucleotide is annealed to a pool of RNA molecules and used as a random primer, so that a great number of cDNA molecules will be generated when extended with reverse transcriptase. Each cDNA would randomly initiate and be extended along the mRNA pool, in principle, until the end of each mRNA molecule. Roche's Primer A is ligated to the 3′ end of the resulting cDNA and the library is amplified. Size selection (200 to 1000 bp) and sequencing the 3′ end of each cDNA will identify the TSS and provide enough sequence information (about 100 nucleotides) to identify to which gene it belongs to. 

**Figure 9:**
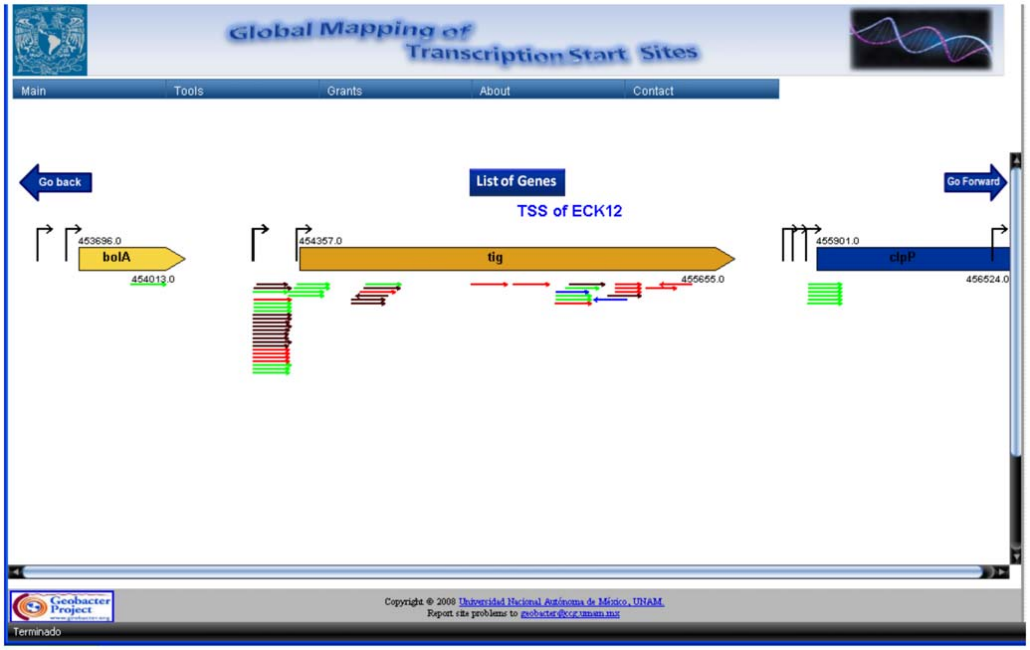
Graphical representation of the *E. coli* chromosome region of the *tig* gene obtained with the GenoSeqGrapher V1.0 program. Each pyrosequencing read is displayed as an arrow below the genomic DNA. Colors represent the different growth conditions from which the sequences were obtained. Mouse over the arrows displays a box with the nucleotide sequence, the position in the genome and the position with respect to ATG of the selected gene.

We prepared cDNA libraries using RNA from four different growth conditions treated to partially remove rRNA, as indicated in Materials and Methods. About 350,000 sequences of *ca* 100 nucleotides long were obtained. Each sequence was aligned to the *E. coli* K12 genome and those corresponding to rRNA operons were culled. We developed a program to be able to analyze this great volume of sequencing data, named GenoSeqGrapher V 1.0 (Taboada, B. *et al*, manuscript in preparation) that graphically displays each sequence below their corresponding position in the *E coli* genome, so that it is very easy to detect where the cDNA ends are located in the genome (Figure 9).

### Robustness of the HTPS Methodology for Genomic TSSs Mapping

Once the rRNA sequences were removed, we ended up with more than 33,000 sequences (even when we treated total RNA to get rid of rRNA, a large proportion of the sequences were still from these RNA species). We realigned each sequence to the *E. coli* genome and clustered together the sequences that ended at the same 3′ end. Eliminating redundancy (multiple sequences pointing to the same 5′ mRNA end), our data set contained 13,181 unique 3′ cDNA ends. Next, we analyzed the sequences corresponding to genes with previously determined TSSs, both from the literature and our DMTSS methodology described above, to evaluate the robustness of the HTPS methodology. We observed the same or very close initiation sites for 225 3′ cDNA ends; 133 of them (59%) mapped exactly or within one nucleotide, 67 (30%) mapped between 2 to 4 nucleotides, while only 25 (11%) mapped with up to 7 nucleotides difference to the previously reported TSS. TSSs mapped further apart where considered products of different promoters. The exact coincidence of the TSSs mapped by DMTSS and HTPS (56 TSSs) was 67%, while 64% of the previously mapped by other methodologies (121 TSSs) coincided precisely to the HTPS data, confirming the precision of our DMTSS methodology. These results indicate that the HTPS strategy developed here is robust and can be used to map TSSs.

### TSSs Detected by HTPS

The 3′ cDNA ends detected here can either be the result of *bona fide* TSSs or of mRNA degradation processes. If multiple sequences pointing to the same TSS were obtained it is more likely that they represented authentic TSSs rather than random mRNA degradation products. Furthermore, if they were from more than one growth condition, the probability of being *bona fide* TSSs increases. Alternatively, they could represent very specific mRNA processing products. Thus, a way to discern between these possibilities is to associate the 5′ mRNA ends to promoter DNA sequences. We searched in the DNA region immediately upstream of each 3′ cDNA end for promoter elements recognized by E-σ^70^ and E-σ^38^ using the strategy described above. Promoter elements with highly significant scores (see Materials and Methods) were detected for 1222 unique 3′ cDNA ends, associated to 906 different genes, 202 TSSs had both −35 and −10 recognition elements for E-σ^70^; 475 had the −10 element only, and 424 with −35 element only. Additionally, the −10 recognition element for E-σ^38^ was detected for 121 more. These promoter elements were located upstream [76.3% (932)], within [14% (171)], or in antisense [9.7% (119)] orientation of the coding region (table 4). As discussed above, it is common to detect TSSs within coding regions. However, it is interesting that a significant proportion of transcripts with promoter sequences are in antisense orientation. It will be critical to study these events and to see if they have any regulatory function.

**Table 4:** Promoters identified by HTPS.

HTPS approach	Total	5′ RACE Evidence	Other Evidence	New Data
−10 element σ^70^	475	39	68	369
−35 element σ^70^	424	10	22	392
−10/−35 elements σ^70^	202	25	40	137
−10 element σ^38^	121	5	7	109
Total	1222	79	137	1007

We also identified 237 additional putative TSSs with no identifiable promoter elements. It is likely that they represent authentic TSSs because they were located upstream of 175 different genes and the same 5′ end was observed in at least two sequences obtained independently, and in many cases from different growth conditions. They could be transcribed from weak promoters or by forms of the RNAP with other σ factors not analyzed here. In conclusion, we identified 1457 putative TSSs with promoter elements or with multiple identical sequences upstream of the coding region.

2079 additional 3′ cDNA ends, with two or more identical sequences obtained independently, were detected within the coding region of 854 genes without obvious σ^70^ or σ^38^ promoter elements. 1541 (74%) were in the same orientation than the ORF, while 538 (26%) were in antisense orientation. Although we have detected *bona fide* TSSs within the coding regions, the lack of recognizable promoter elements in front of these sequences precluded us to call these 3′ cDNA ends TSSs until we have additional supporting information.

**Figure 10:**
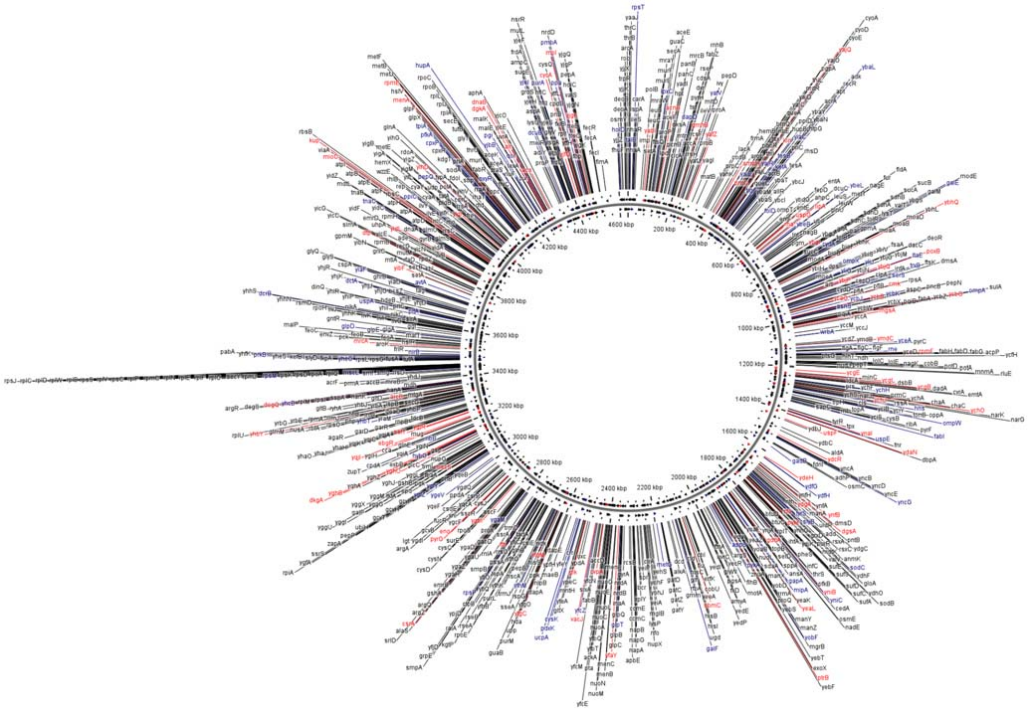
Display on the *E. coli* K-12 chromosome of all the TSSs obtained in this work by DMTSS (red) and by HTPS (black). TSSs obtained by both methodologies are shown in blue.

The unbiased results of the HTPS revealed a great number of transcripts in antisense orientation. Even when it is uncertain that the 5′ of the antisense sequences detected here are the result of transcription initiation processes, the fact that they are not distributed randomly suggests that they represent actual transcription events of the cell. They may be involved in gene expression control by duplex formation of their complementary transcripts, as detected in several other systems [42]. All the TSSs detected by pyrosequencing with an associated promoter are shown in Table S1, and graphically displayed in Figure 10.

### Specificity of the Initiation Process

**Figure 11:**
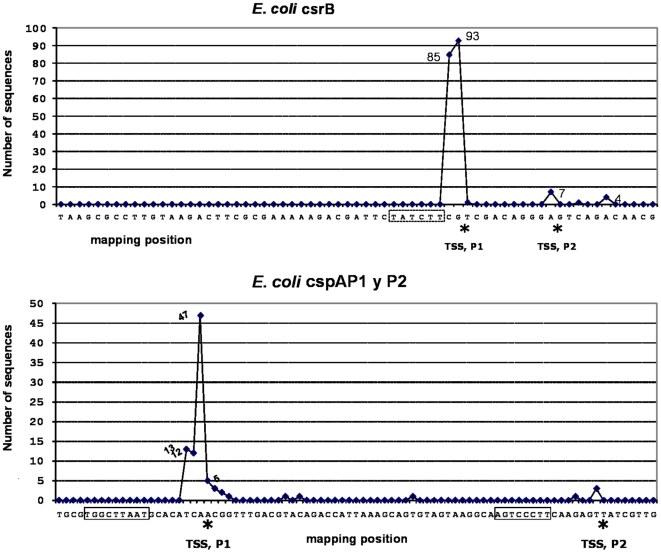
Multiple TSSs for a single TU. The graph shows several sequences upstream of the *csrB* and *cspA* genes initiating at different positions, showing the ambiguity of the TSS in some TU.

Previous methodologies used to identify TSSs did not quantify the frequency of initiation at each position in respect to the promoter, as the TSS was simply taken as the major band in a gel. The advantage of the HTPS method is that each sequence represents a single transcription event, so that it is now possible to quantify the relative number of transcriptions initiation events. The high redundancy of data obtained by HTPS for highly expressed genes, allowed us to observe that the transcription initiation site is not an exact location downstream of the promoter. Figure 11 shows the variation of TSSs location for *csrB* and *cspA*. The 178 sequences obtained from *csrB*P1 initiate at two adjacent nucleotides almost in equal proportions, while *cspA* is more variable, having a preferred site but using three other adjacent sites. In this context, it seems that the +1 position should rather be defined as the site where the majority of the transcription events initiate, although the RNA polymerase actually initiates around this site. It is likely that the RNA polymerase docks to different promoter regions with different strengths, so that some initiate in a more relaxed form than others, resembling message slippage during elongation. These results indicate that HTPS for TSS mapping also provides relevant data to understand the basic mechanisms of transcription initiation. It will be interesting to relate different promoter properties to the strictness of the TSS.

### Frequency of Each Initiation Nucleotide

**Figure 12:**
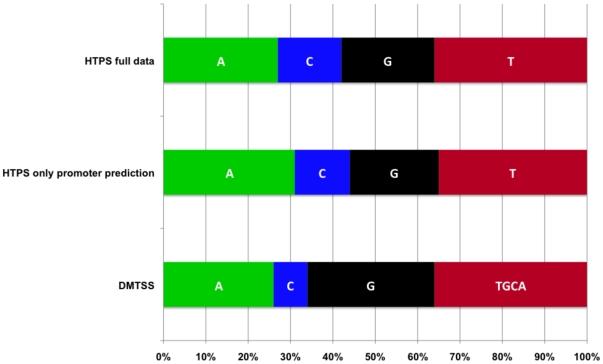
Frequency of each initiation nucleotide. The graph shows the frecuency of the starting nucleotide (adenine, guanine, cytosine and thymine) TSSs obtained by DMTSS, by HTPS, and for the TSSs with predicted promoters from the HTPS data set. AGCT in DMTSS indicates any nucleotide, see text.

With our dataset of more than 1700 TSSs detected by DMTSS and by HTPS, we calculated the frequency at which each nucleotide initiated transcription. As shown in Figure 12, for the TSSs identified by DMTSS, 30% initiated with guanine, whereas 26% started with adenine. The least utilized initiator nucleotide was cytosine, which was used in roughly 8% of transcripts. Mainly due to the ambiguity generated by the polyadenine tailing, we were unable to unambiguously determine the precise nucleotide type used to initiate transcription for the remaining 36% of the transcripts (labeled as ATGC in Figure 12). For TSSs identified by HTPS with promoter prediction, thymine was the most common initiating nucleotide (35%) while adenine was used in 31% of the cases. The nucleotide least used was also cytosine (12%). In conclusion, purines were more frequently utilized as initiating nucleotides.

### Prediction Regulatory TF Binding Sites

In order to evaluate the presence of putative binding sites for transcriptional factors (TFs), which can certainly help to better understand transcriptional regulation of the newly mapped promoters, we screened the regions around each TSS as described in Materials and Methods. 55 different TFs binding sites were detected with high confidence, of which the most frequently found were those for CRP, Fis, PhoB, RhaS, PurR and FNR (Table S4 and Figure S6), of which, CRP, Fis and FNR are general TFs that regulate many genes in *E. coli* (Table S5). It is noteworthy that binding sites for ArcA, the transcriptional regulator expressed under low oxygen conditions, were only detected in a single region, reflecting perhaps that we only screened TSSs from aerobic cultures. Table S4 shows the genes for which at least one TF binding site was detected.

**Figure 13:**
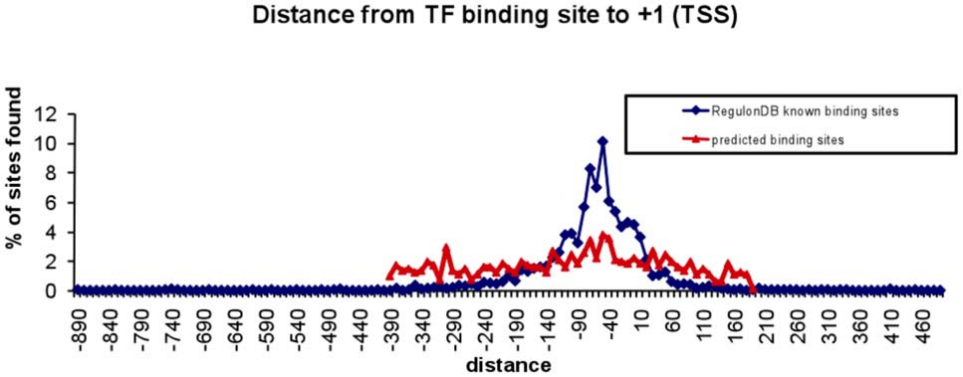
Distance of the predicted TF binding sites to the TSSs described in Table S1. Data obtained in this work were compared with that of RegulonDB.

The distance between the TSSs and the putative TF binding sites is shown in Figure 13. The distribution is similar to the one observed for the experimentally determined TF binding sites, thus, it is highly likely that the sequences reported here are *bona fide* TF binding sites.

### Length of the Untranslated 5′ Region

**Figure 14:**
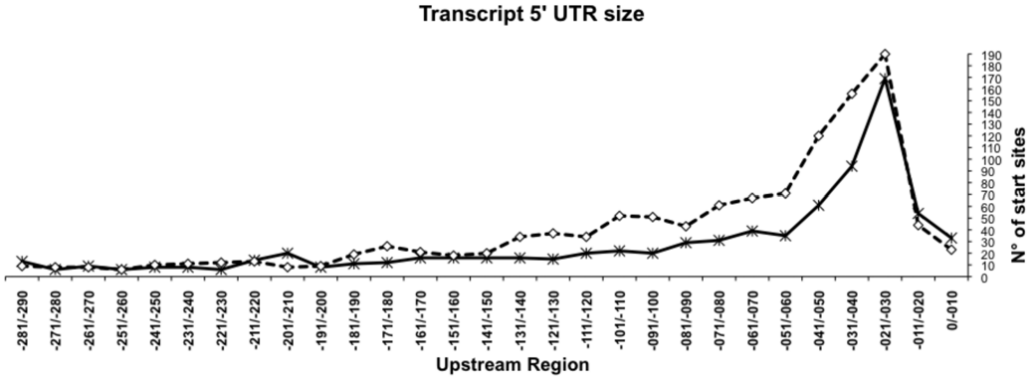
Length of the 5′ untranslated region (5′ UTR). The distances of each TSS mapped to the ATG translation initiation codon is plotted (5′ UTR). Dataset obtained in this work (solid line), and in all the previously mapped TSSs in RegulonDB (dashed line). For both data sets the most frequent 5′ UTR length was between 20 to 40 nucleotides.

Next, we determined the 5′ UTR length (from the TSS to the first ATG codon of the product) for each of the about 1000 TUs located upstream of ATG. Figure 14 shows that the spacing between the TSSs and the predicted translation start codons mostly varies between 20 and 40 nucleotides. Very few TSSs were shorter than 20 nucleotides, while some have a long 5′ UTR (between 100 and 290 nucleotides upstream of the translation start codon). These results are also in agreement with previously reported analyses of other TSSs in *E. coli* adding further support to proper TSS determination by our genome-wide methodology [43]. The latter transcripts, by virtue of having a large stretch of untranslated RNA, might possibly contain conserved regulatory RNA elements, such as sRNA or riboswitches regulated by metabolites. We searched in the Riboswitch Explorer, a database that contains all the current information on genes with experimentally verified riboswitches across phylogenetically distant organism [44] for matches with known regulatory RNAs. Nine genes whose TSS were identified in this work, *thrA*, *ybaB*, *rib*, *alx*, *lysC*, *thiM, ribD*, *rpsO* and *rpsM*, with a long 5′ UTR, very probably have a riboswitch or another RNA regulatory element.

### Identification of Attenuators in the Untranslated 5′ Region

Attenuators are regulatory RNA structures that modify gene expression by altering transcription or translation. Transcriptional attenuators require the formation of one of two mutually exclusive RNA-secondary structures in the leader sequence of a transcript, the terminator and the antiterminator. Antiterminators are RNA structures that block the formation of terminators and make possible the transcription of downstream genes, while terminators block transcription of these genes when the products are not necessary. A translational attenuator is also an RNA structure that works by blocking, by base pair complementary, the Shine-Dalgarno ribosomal-binding motif required to properly initiate translation [45]. Therefore, RNA secondary structures downstream of a TSS can drastically affect transcription and/or translation efficiency.

We searched for the known attenuator elements, reported by Merino and Yanofsky [45], between the TSS and the translation initiation codon in each of the 1222 TSSs associated to σ^70^ and σ^38^ promoters, and also in the 237 TSSs upstream of ORFs for which we did not detect promoter elements with high confidence. We found 76 attenuator sequences, almost half of them translational attenuators (Table S6). Other attenuators reported by Merino and Yanofski [45] that were not detected here, very likely are from genes expressed in conditions different to the ones used here. In conclusion, in addition to the promoter elements and TF binding sites, we were able to identify structural elements such as attenuators and terminators in the untranslated leader region of several genes.

We have been gathering information from the primary literature about the regulation of gene expression in *E.coli* for at least 10 years, when the first RegulonDB version was published [46]. Here we started with “active annotation”, that is, following experimental approaches to gather missing information on regulatory elements. In this report, we implemented two different methodologies, DMTSS and HTPS, designed for genome-wide TSSs mapping. Using these strategies, we solved more than 1700 TSSs (Figure 10) and identified the most likely promoter sequences, the type of σ factors and in some cases putative regulatory binding sites for transcriptional regulators associated to them. These results are the first large-scale efforts to accurately map TSSs and identify promoter and regulatory elements controlling gene expression in *E. coli.* These “active annotation” efforts have considerably increased the number of known TSSs in the *E. coli* genome. We would like to point out that even when we did not map 5′ triphosphate ends, the ultimate proof of transcription initiation events, we have solid experimental evidence for more than 2400 TSSs. We are currently analyzing other growth conditions in order to have TSSs for each TU of *E. coli*, this will certainly help to understand the regulation of each gene and TU in the genome. In addition to the intrinsic contribution to the regulatory mechanisms of gene regulation, the identification of TSSs is also useful to improve the prediction of gene regulation, not only in terms of which TF control their expression, but also potentially the precise activator or repressor role of each TF on promoters. A second layer of complexity is one of multiple TSSs and, in some cases, different types of sigma factor governing expression of a single gene or TU.

RegulonDB provides the largest electronically encoded regulatory network of any free-living organism. The combined strategy of literature curation and now of experimental high-throughput characterization of the regulatory network is contributing towards what may be the first comprehensively annotated genome, the one of *Escherichia coli* K-12.

## Materials and Methods

### Targeting of transcriptional units for TSS determination based on DMTSS

The *E. coli* RegulonDB database version 5.6 was used as a bioinformatics tool to search and select for those TUs with currently unknown experimentally assigned TSSs and promoter sequences. We ordered TUs according to their level of expression based on three different microarray gene-expression profiles data sets [47], [48], and the most highly expressed in LB and M9 minimal growth media were selected to increase the chance of obtaining their corresponding mRNA (Figure 1A).

### Design of gene-specific primers for PCR amplification and sequencing reactions

Based on TUs information in RegulonDB, including both experimentally determined and predicted, the first gene of each TU was selected as the target to perform primer-specific PCR amplifications and sequence analyses (see details below) with the aim of precisely mapping as many TSSs as possible. Accurate PCR amplification of the individual genes from the cDNA library was favored by designing primers, which allow specific annealing with the non-template strand of its own target gene. For this purpose, we developed software called “Genome Primer Analysis” (GPA) in which the following criteria were followed to achieve optimal primer design: oligonucleotide length, G+C content, and minimizing palindromic sequence formation. Candidate primer sites were evaluated within a gene coding-sequence window between 100 to 300 nucleotides downstream of the translation start codon, taking into account how many consecutive nucleotides at the 3′ end of each primer were present elsewhere in the *E. coli* genome. Based on these considerations the program designs an array of primers for each gene that fulfill these criteria. In summary, the primers did not exceed 20 nucleotides in length; the G+C content was between 55 to 65% with a melting temperature (Tm) around 60 to 64°C, and most importantly, for each primer the last eight nucleotides of the 3′ end region were unique within the *E. coli* genome. Finally, from the collection of output primers generated by the GPA program we selected the best one for each gene, by imposing another stringent filter, which consisted in that the sequence of the last 6 nucleotides of the 3′ region of the desired primer should not be found in the highly expressed 16S and 23S ribosomal genes. This criterion was followed after we detected several products that originated from rRNAs instead of the specific gene for which the primers were designed, due to local 3′ match of eight or more nucleotides (Figure 1A).

### DMTSS approach for TSS identification

We used as experimental platform the 5′ RACE methodology [20], [21] to map the TSSs of the selected TU, with some modifications. Figure 1B shows the complete strategy for genome-wide TSSs mapping, making emphasis in the modifications made to the 5′ RACE. We named this genomic strategy DMTSS.

#### Extraction of total RNA

Total RNA from the wild type *E. coli* MG1665 (K-12) strain was purified from three different growth conditions: (1) Luria Broth (LB), 30°C, OD 600 nm 0.8; (2) Minimal Medium (M9) supplemented with 0.2% glucose as the solely carbon source, 30°C, OD 600 nm 0.8; and (3) M9 supplemented with 0.2% glycerol, 30°C, OD 600 nm 0.5 was isolated. These growth conditions exactly reproduced the conditions used in previous gene expression profile microarray experiments obtained for this *E. coli* strain [47], [48]. Briefly, 1 ml of the culture at the desired growth level was centrifuged to collect the cells, and the RNA was isolated using the RNeasy Mini kit, Qiagen (Valencia, CA.) according to the manufacturer's instructions and checked by agarose gel electrophoresis.

#### Generation of cDNA libraries

At this step, and contrasting to the standard 5′ RACE protocol that uses a specific primer per gene, DMTSS employs a random hexamer primer to generate cDNA products for each RNA source. Briefly, 3 µl (approximately 1.5 µg) of purified RNA sample was mixed with 700 pmol of random primer at 70°C for 10 minutes. The reaction was cooled on ice for 5 min, and immediately a reaction mix containing: 5 µl of dNTPs (2.5 mM), 1 µl of DTT, 4 µl of 5X reaction buffer and 1 µl of SuperScript III reverse transcriptase (200 U/µl) in a 20/µl final volume was added. All these reagents were purchased from Invitrogen (Carlsbad, CA). To allow cDNA synthesis the reactions were incubated in a Stratagene RoboCycler PCR instrument (Amsterdam, The Netherlands) under the following program: (28°C for 20 min, 45°C for 40 min, 70°C for 10 min). The final cDNA product was purified using the Roche's High Pure PCR Product Purification Kit (Indianapolis, IN), according to the manufacturer's instructions.

#### cDNA labeling with an homopolynucleotide tail

The purified cDNA libraries were enzymatically labeled at the 3′ terminal end with a homo polynucleotide tail. Briefly, a 20 µl purified cDNA sample was mixed with 1 µl of Terminal Deoxynucleotidyl Transferase (20 U/µl), 0.2 mM final concentration of the appropriate dNTP and 1X reaction buffer in a 25 µl final reaction volume. The reagents were purchased from Fermentas (St. Leon-Rot,. Germany). The reaction was incubated at 37°C for 30 min, following by enzyme inactivation by heating at 70°C for 10 min.

#### Linear amplification of tagged cDNA library

Next, and differing from the standard 5′ RACE protocol, we performed a linear PCR amplification in order to enrich the yield of the cDNA complementary strand. Briefly, 20 pmol of DMTSS-1 primer (5′-GAC-TCG-AGT-CGA-CAT-CGA-NNN-NNN-NNN-NNN-NNN-NN-3′; N is the complementary nucleotide to the homopolymer tail), which has an adaptor sequence at its 5′ terminal end (underlined), was allowed to anneal to the poly homonucleotide tract of the tagged cDNA, and used to linearly expand the library in a standard PCR amplification reaction under the following conditions: 1 cycle, 94°C for 10 min; 30°cycles of 94°C for 1 min, 45°C for 2 min, 72°C for 3 min, and finally one last extension cycle at 72°C for 5 min).

#### Primer-specific PCR amplification and sequence reaction for TSS identification

Finally, the cDNA pool was used as template to selectively and individually amplify each gene or TU by Hot-start PCR. This was achieved by using a DMTSS-4 primer, common to all reactions (5′-GAC-TCG-AGT-CGA-CAT-CGA-TT-3′), which carry the adaptor sequence and a primer that specifically anneal with the cDNA complementary strand of their target gene (see Figure 1B). A sample of the PCR product was analyzed by 8% polyacrylamide gel electrophoresis (PAGE) and the band or bands obtained, if more than one TSS were being produced, were excised and purified from the gel. Finally, the purified PCR products were sequenced using the same specific gene-primer employed for PCR amplification. Sequence reactions were done in an Applied Biosystems 3100 Genetic Analyzer/ABI PRISM device. The sequences were aligned with the *E. coli* K-12 genome and the putative TSSs were identified as the first nucleotide immediately adjacent to the polynucleotide tail.

### HTPS approach for TSS identification

Total RNA from *E. coli* K12 grown in LB and minimal media at both 37°C and 30°C was extracted and the rRNA was eliminated using the MicrobExpress kit (Ambion). We generated cDNA libraries by reverse transcription using SuperScript III reverse transcriptase together with an hexamer random primer-adaptor B (5′GCCTTGCCAGCCCGCTCANNNNNN3′). The cDNA synthesis reactions were incubated in a RoboCycler equipment (Stratagene, Amsterdan, The Netherlands) under the following program: 28°C for 20 min, 45°C for 40 min, 70°C for 10 min. The cDNA final products were purified using the High Pure PCR product purification kit (Roche Indianapolis, USA), according to the manufacturer instructions. A double stranded adaptor A was ligated to purified cDNA libraries (tagged). One of the oligonucleotides of this double stranded adaptor has a randomized sequence of six nucleotides that match with the 3′ end of the cDNA products.

5′GCCTCCCTCGCGCCATCAGNNNNNN3′

3′CGGAGGGAGCGCGGTAGTC5′

5 µl of purified cDNA sample was mixed with T4 DNA ligase (1 Weiss U/µl), 1X reaction buffer, 35 pmol of adaptor A, in a final reaction volume of 25 µl. The reaction was incubated at 16°C overnight, following by enzyme inactivation at 70°C for 10 minutes. All the reagents were purchased from Fermentas (St. Leon-Rot, Germany).

Using primers complementary to adaptors A (5′GCCTCCCTCGCGCCATCAG3′) and B (5′GCCTTGCCAGCCCGCTC3′), PCR amplicons were generated by Fast Start High Fidelity PCR System (Roche Applied Sciences, Indianapolis, USA), purified with MiniElute PCR purification Kit (Qiagen, Valencia, USA), and quantified using the NanoDrop spectrophotometer (NanoDrop Technologies, Wilmington, USA). At least 3 µg of cDNA were obtained for each sample. The quality of the DNA was evaluated by capillary electrophoresis using the Agilent Bioanalyser 2100 (Agilent Technologies, Palo Alto, USA). For pyrosequencing, samples were prepared according to 454 Roche GS20 DNA Amplicon Library Preparation Kit user manual. Each amplicon mix was sequenced independently using the GS emPCR Kit II (454 Life Sciences Corporation, Branford, USA). The sequence was done at Laboratorio Nacional de Genomica para la Biodiversidad (LANGEBIO) Cinvestav, Irapuato, México.

### Promoter prediction based in WCONSENSUS and PATSER programs

We made use of one of the 12 highest PWMs described by Huerta and Collado-Vides [12] for the σ^70^. This matrix was chosen based on its high discrimination of the −10 and −35 elements [12]. With the PATSER program (ftp://www.genetics.wustl.edu/pub/stormo/Consensus/ [39], which searches for patterns in a database, and the sequences utilized in this work for the construction of the PWM, the following thresholds were defined: −0.5 SD for the −10 element, and −1 SD for the −35 element. The threshold for the −10 element was chosen because with it, 60% of the promoters used for the training set were recovered. Because the −35 element is less conserved than the −10 element, a lower threshold was selected to avoid false positives. With this value, 40% of the promoters of the initial training set were recovered. For the σ^38^ −10 element a new matrix was constructed with the WCONSENSUS program [39], utilizing the nucleotide sequences of 71 promoters of *E. coli* annotated in RegulonDB. For this purpose, with a threshold identical to the one used for the −10 element of σ^70^, we recovered 65% of the promoters of the training set.

With the thresholds mentioned above, PATSER was used in order to search for the highest scores within the 60 nucleotides upstream of each of the unique 5′ ends (13,181). For the −10 region, we selected those results where the search pattern was located within the first 20 nucleotides upstream of the 5′ end. We choose this distance because it is well known that the −10 element for σ^70^ is located −4 to −12 nucleotides from the TSS [49]. For the −35 region the searches were done in the same manner that for the −10 region, but taking into account also the distance of the −10 element (−4, −12 bases) and the spacing distance between both elements (15 to 21 nucleotides) [29]. This allowed us to eliminate sequences where the −35 element was located in awkward positions.

### TFs binding sites predictions

In order to predict regulatory sites, the regions from −400 to +200 of each TSS were scanned for the known TFs binding sites annotated in RegulonDB (recall that TF binding sites are located within this window), using the program Matrix-Scan [50]. The weight matrices described RegulonDB for TFs binding sites, which were evaluated by their quality using the method described by Medina-Rivera et al (in preparation), were used for this purpose.

## Supporting Information

Figure S1 Electropherograms from multiple experiments of TSS mapping for the *rpsP* gene. All the sequences pointed to the same TSS that is identical to the reported [53], indicating that the DMTSS is a very robust method for mapping initiation events.
(0.58 MB PDF)

Figure S2 Experimental results of TSS mapping for three different genes showing the PCR product(s), electropherogram, and the DNA alignment with *E. coli* K12. a) gene *pepN*, b) gene *ydaN*, and c) gene *gdhA*.
(0.96 MB PDF)

Figure S3 Unspecific priming of oligonucleotide ybbA into *gdhA* and *sstT* genes. A) PCR products obtained with oligonucleotide ybbA, designed for *ybbA* gene. B) Partial base-pair complementarily of this oligonucleotide with *gdhA* and *sstT* regions. Products 1 and 2 correspond to the upstream region of *gdhA*, while product 3 corresponds to the upstream region of *sstT*. No product corresponding to gene *ybbA*, for which the oligonucleotide was designed, was detected.
(0.17 MB PDF)

Figure S4 Solving the TSS ambiguity by using a different polynucleotide for the 3′ end labeling. In the case of the *ydfH* gene, the ambiguity was for only one nucleotide (adenine or guanine). By using a different nucleotide for tailing, for instance thymine instead of adenine, as we did in this case, the ambiguity is solved. This change shows that the guanine nucleotide was indeed the TSS of the *ydfH* gene under the conditions tested. A) Incorporation of dTTP at the 3′ end of the cDNA. B) Incorporation of dATP at the 3′ end of the cDNA.
(0.35 MB PDF)

Figure S5 Gene context of the *csrB*, *tff* and *sroG* small RNAs.
(0.11 MB PDF)

Figure S6 Location of the top TF's binding sites predicted in the regulatory region of each TU with promoter prediction. The complete data set is stored at http://www.ccg.unam.mx/Computational_Genomics/SupMaterial/TSS/index.html
(0.11 MB PDF)

Table S1 Results for prediction of −10 and −35 elements recognized by σ^70^ and σ^38^ with the program PATSER [39].
(2.63 MB XLS)

Table S2 Oligonucleotides utilized for DMTSS.
(0.22 MB XLS)

Table S3 Genes with known, predicted, conserved, putative, hypothetical or unknown function.
(0.07 MB XLS)

Table S4 Putative binding sites for 55 transcription factors were searched in the regions from −400 to +200 for each TSS with promoter prediction, using the Matriz-Scan program.
(0.16 MB XLS)

Table S5 Transcriptional factors binding sites associated to each TSS. The information in RegulonDB for each of these TFs and their binding sites is shown.
(0.05 MB DOC)

Table S6 Genes with associated attenuator identified in this work.
(0.04 MB XLS)
