# Supplementary figures and images for: Genome-Wide Identification of Transcription Start Sites, Promoters and Transcription Factor Binding Sites in E. coli
Source: PLoS One. 2009 Oct 19;4(10):e7526. doi: 10.1371/journal.pone.0007526 (PMC2760140; doi:10.1371/journal.pone.0007526)

Figure S1

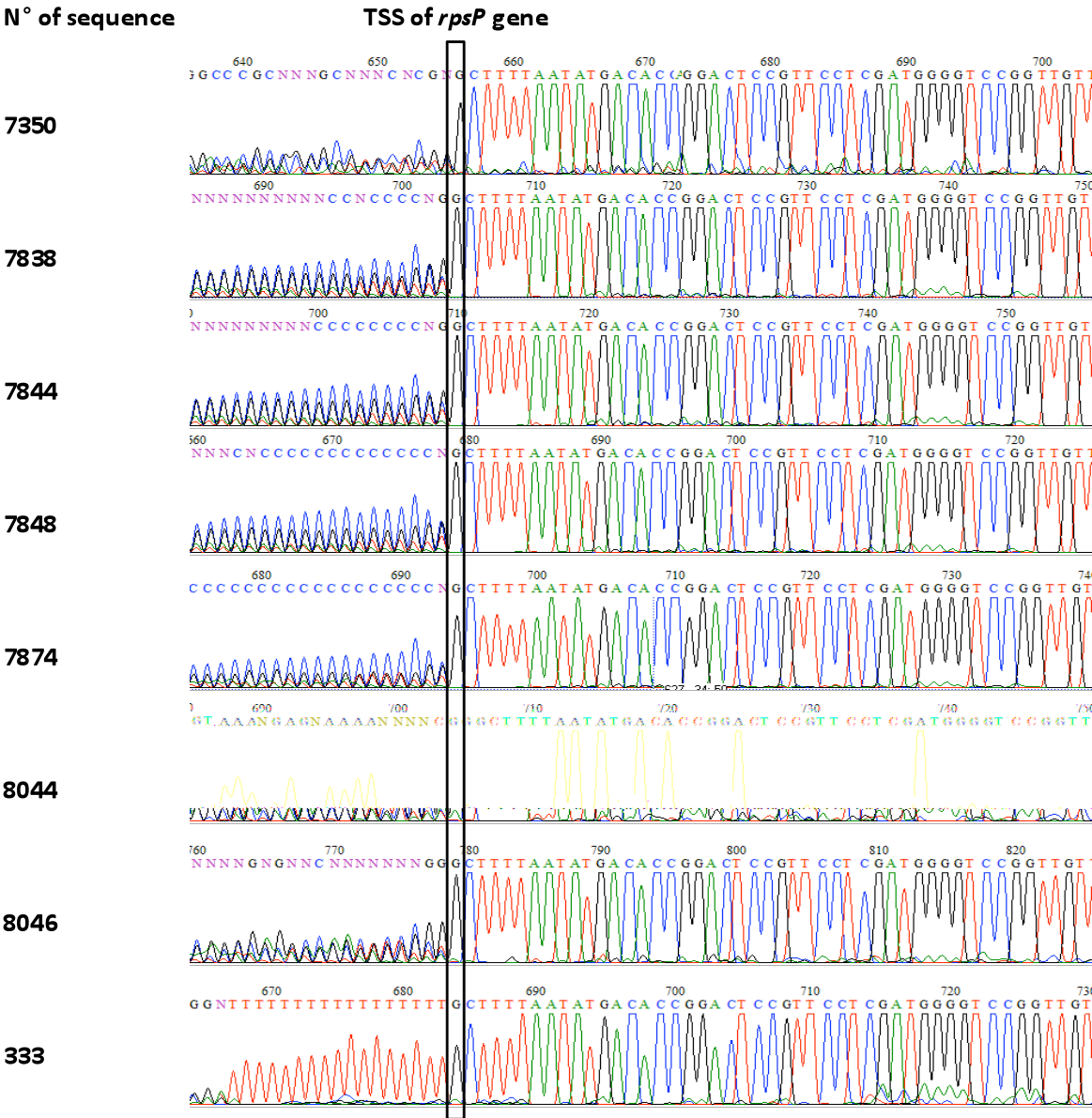

Supplement: Figure S1 — Electropherograms from multiple experiments of TSS mapping for the rpsP gene. All the sequences pointed to the same TSS that is identical to the reported [53], indicating that the DMTSS is a very robust method for mapping initiation events. (0.58 MB PDF) [file pone.0007526.s001.pdf]

Figure S3

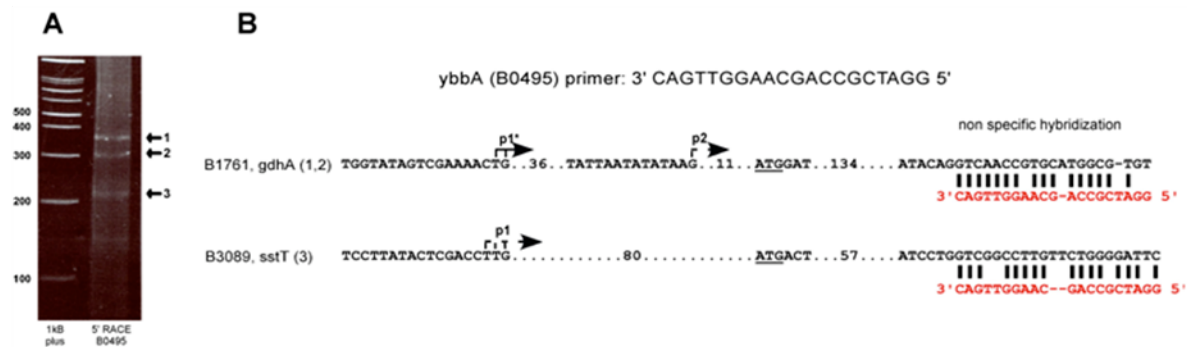

Supplement: Figure S3 — Unspecific priming of oligonucleotide ybbA into gdhA and sstT genes. A) PCR products obtained with oligonucleotide ybbA, designed for ybbA gene. B) Partial base-pair complementarily of this oligonucleotide with gdhA and sstT regions. Products 1 and 2 correspond to the upstream region of gdhA, while product 3 corresponds to the upstream region of sstT. No product corresponding to gene ybbA, for which the oligonucleotide was designed, was detected. (0.17 MB PDF) [file pone.0007526.s003.pdf]

Figure S4

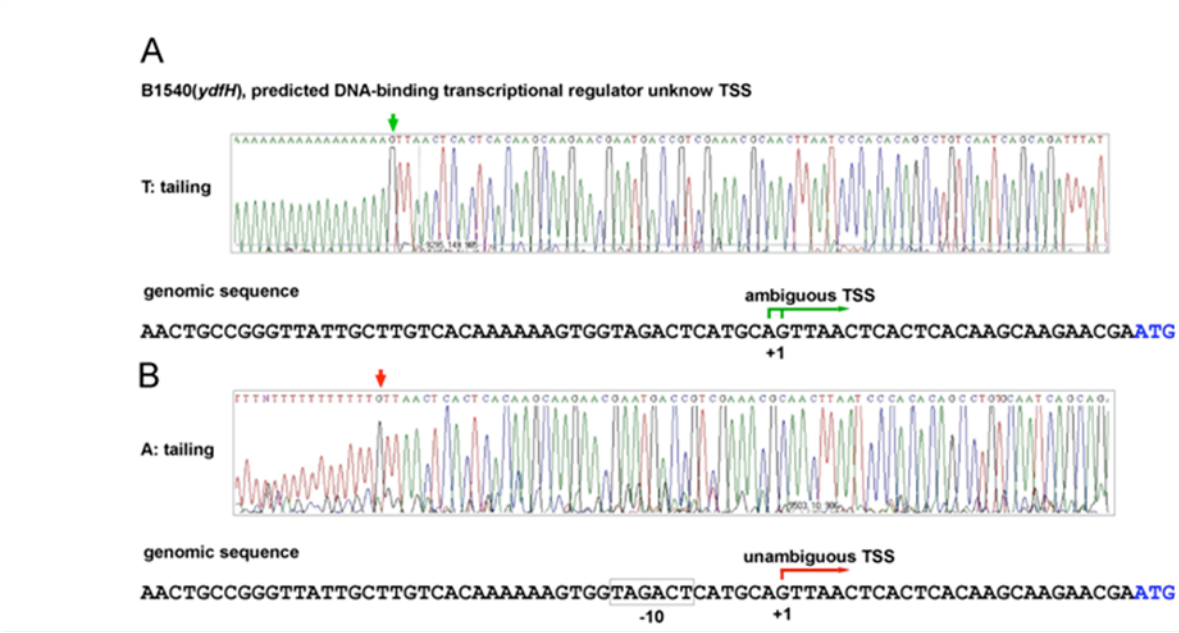

Supplement: Figure S4 — Solving the TSS ambiguity by using a different polynucleotide for the 3′ end labeling. In the case of the ydfH gene, the ambiguity was for only one nucleotide (adenine or guanine). By using a different nucleotide for tailing, for instance thymine instead of adenine, as we did in this case, the ambiguity is solved. This change shows that the guanine nucleotide was indeed the TSS of the ydfH gene under the conditions tested. A) Incorporation of dTTP at the 3′ end of the cDNA. B) Incorporation of dATP at the 3′ end of the cDNA. (0.35 MB PDF) [file pone.0007526.s004.pdf]

Figure S5

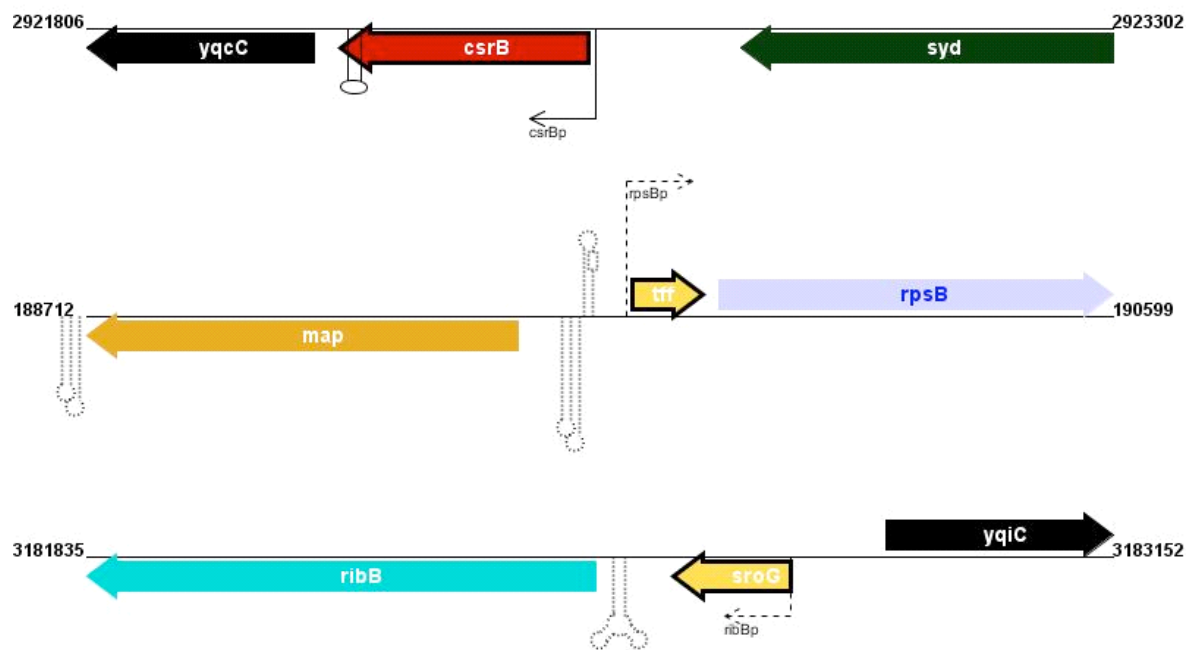

Supplement: Figure S5 — Gene context of the csrB, tff and sroG small RNAs. (0.11 MB PDF) [file pone.0007526.s005.pdf]

Figure S6

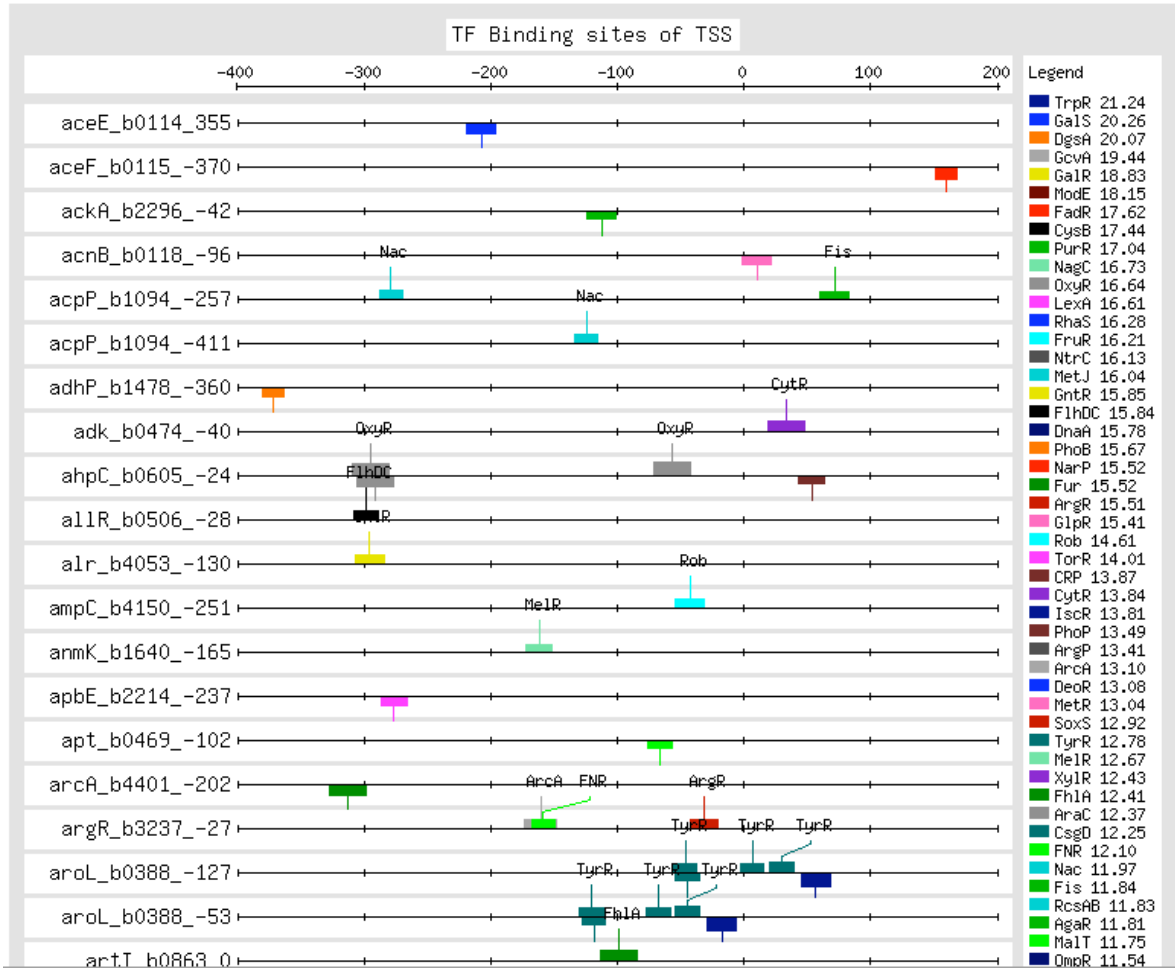

Supplement: Figure S6 — Location of the top TF's binding sites predicted in the regulatory region of each TU with promoter prediction. The complete data set is stored at http://www.ccg.unam.mx/Computational_Genomics/SupMaterial/TSS/index.html (0.11 MB PDF) [file pone.0007526.s006.pdf]
