# Supplementary material for: Genome-Wide Identification of Transcription Start Sites, Promoters and Transcription Factor Binding Sites in E. coli
Source: PLoS One. 2009 Oct 19;4(10):e7526. doi: 10.1371/journal.pone.0007526 (PMC2760140; doi:10.1371/journal.pone.0007526)

Figure S2

a) b3677 *pepN*, Aminopeptidase N

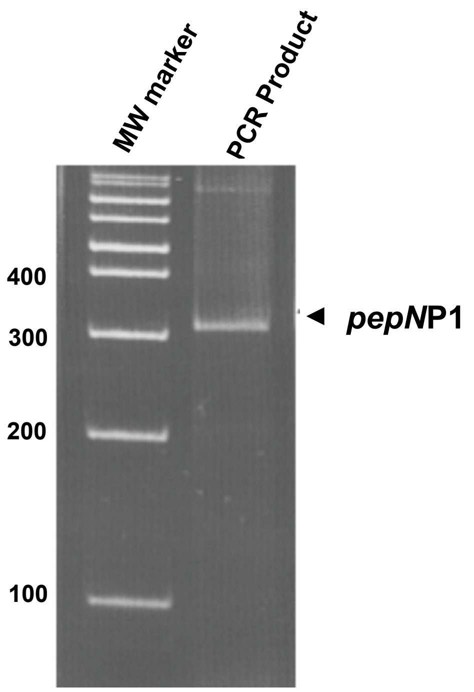

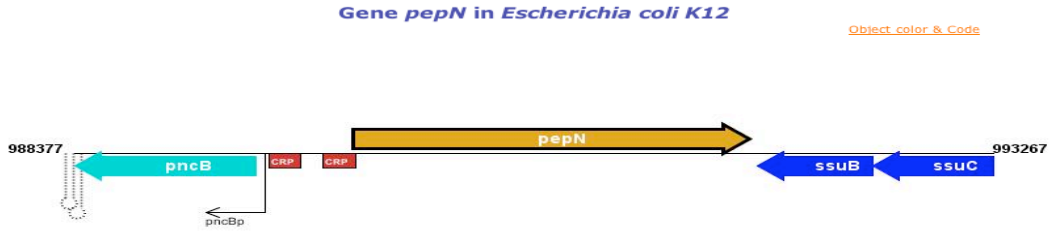

The TSS of the *pepN* gene has not been reported.

*pepN*P1

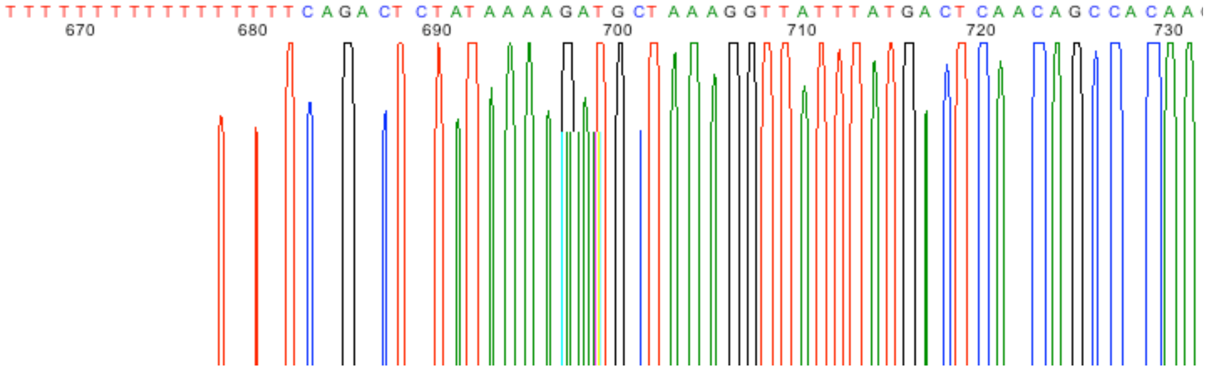

Sequence of the PCR product

```
   Seq-PCR ----------------TTTTTTTTTTTTTTTTTTTCAGACTCTATAAAAGATGCTAAAGGTTATTTATGACTCAACAC
Genomic_seq TAGCGCGATTTACGCAACAGGAATAGACTGAACACCAGACTCTATAAAAGATGCTAAAGGTTATTTATGACTCAACAC
  pepN_ORF ------------------------------------------------------------------ATGACTCAACAC
     ruler ...440.......450.......460.......470.......480.......490.......500.......510..
```

Start codon

Figure S2a

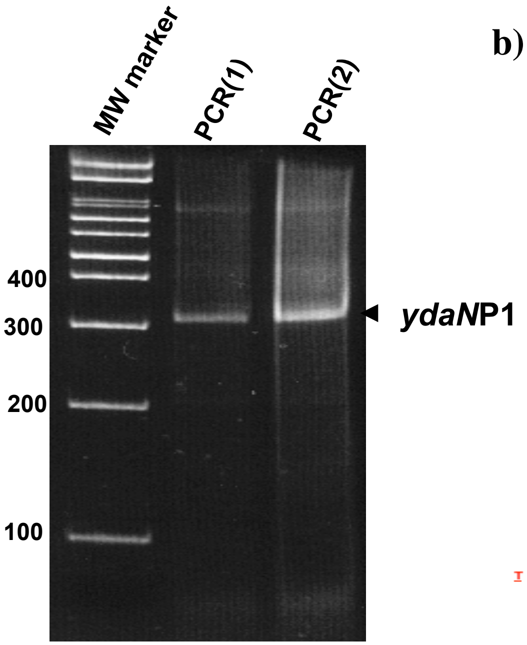

Two independent experiments produced the same results.

b) b1342 *ydaN*, predicted Zn(II) transporter

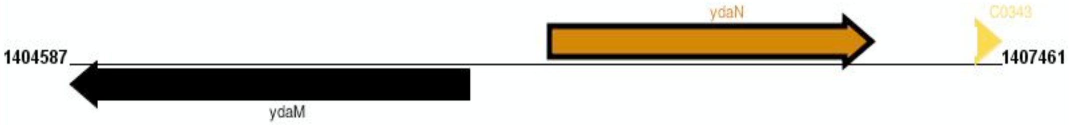

The TSS of the *ydaN* gene has not been reported

*ydaN*P1

Sequence of PCR(1)

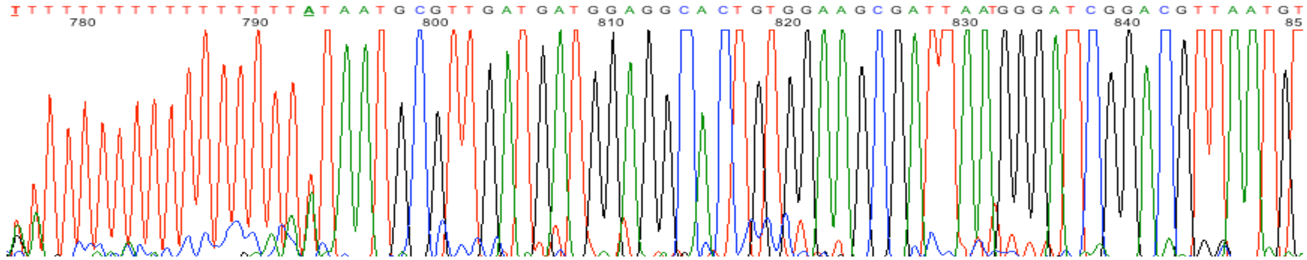

```
Seq-PCR_1_  ------------TTTTTTTTTTTTTTTTTTTATAATGCGTTGATGATGGAGGCACTGTGGAAGCGATTAAGGGATCGGACGTTAA
Genomic-seq GCGTAACAGGGCCAGAAGGCTAGACTACAAAATAATGCGTTGATGATGGAGGCACTGTGGAAGCGATTAAGGGATCGGACGTTAA
Seq-PCR_2_  ------------TTTTTTTTTTTTTTTTTTTATAATGCGTTGATGATGGAGGCACTGTGGAAGCGATTAAGGGATCGGACGTTAA
ydaN-ORF    ---------------------------------------------------------GTGGAAGCGATTAAGGGATCGGACGTTAA
ruler       ...450.......460.......470.......480.......490.......500.......510.......520.......53
```

Start codon

Sequence of PCR(2)

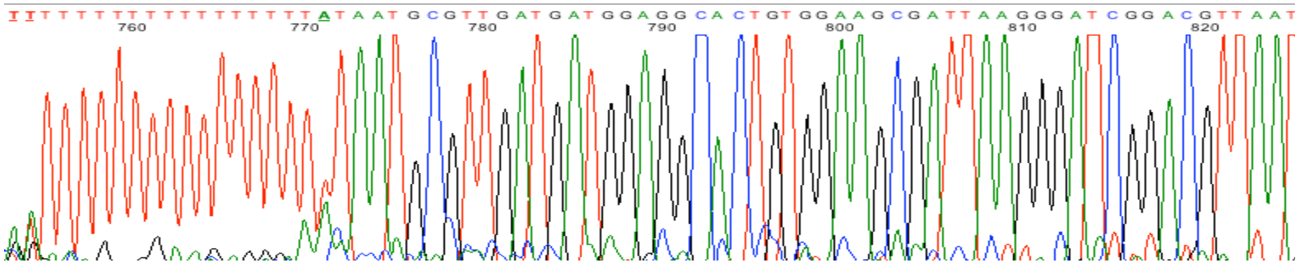

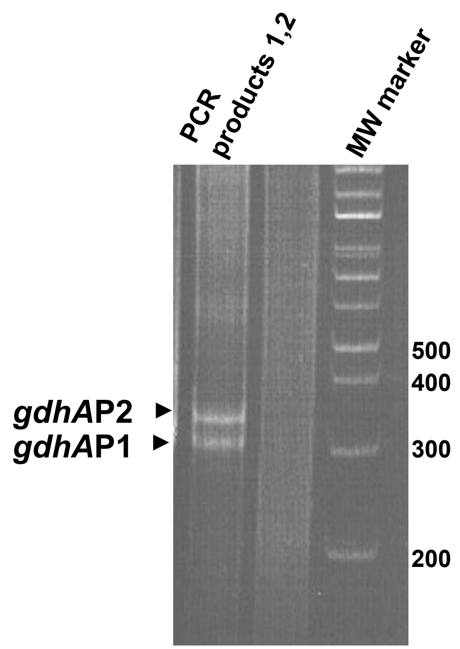

c) b3300 *gdhA* Glutamate dehydrogenase

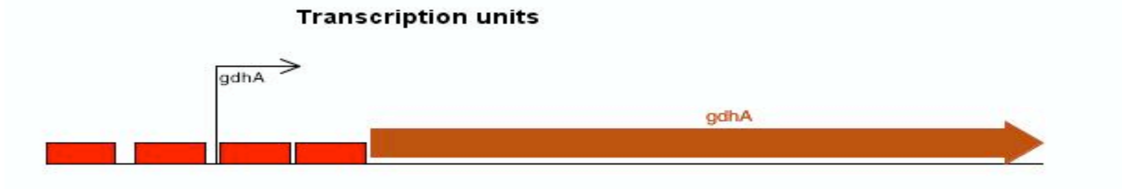

The TSS of the *gdhA* gene is identical to the previously reported.

Name: gdhA
+1: 1840331
Sigma Factor: Sigma70
Sequence: gctttcctgggtcatttttttcttgcttaccgtcacattcttgatggtatagtcgaaaacTgcaaaagc
Reference(s): [1] Riba L., 1988

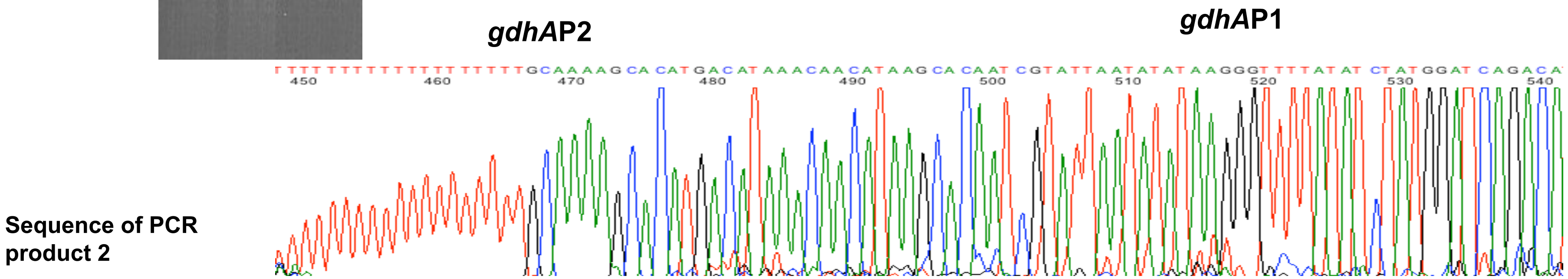


```
     PCR_2  -----------TTTTTTTTTTTTTTTTTGCAAAAGCACATGACATAAACAACATAAGCACAATCGTATTAATATATAAGGGTTTTATATCTATGGATCAGACATA
Genomic_seq CACATTCTTGATGGTATAGTCGAAAACTGCAAAAGCACATGACATAAACAACATAAGCACAATCGTATTAATATATAAGGGTTTTATATCTATGGATCAGACATA
  gdhA-ORF  -------------------------------------------------------------------------------------------ATGGATCAGACATA
     PCR_1  -------------------------------------------------------------TTTTTTTTTTTTTTTTTTGGTTTTATATCTATGGATCAGACATA
     ruler  0.......420.......430.......440.......450.......460.......470.......480.......490.......500.......510....
```

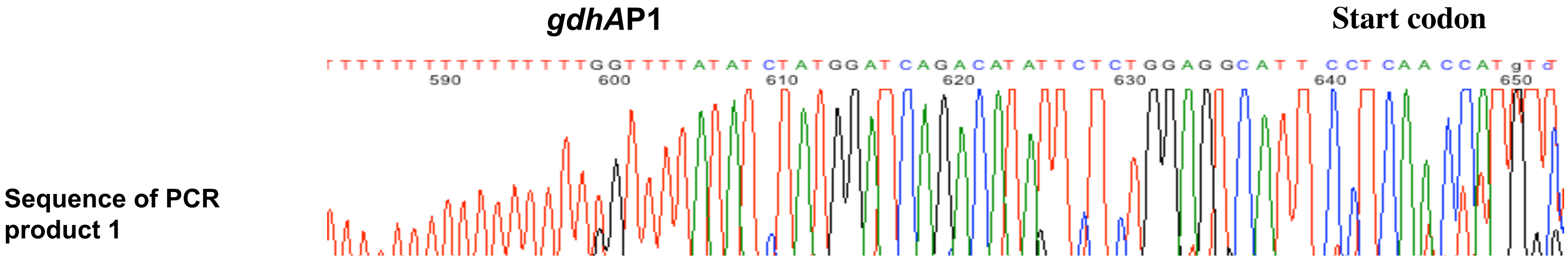

Supplement: Figure S2 — Experimental results of TSS mapping for three different genes showing the PCR product(s), electropherogram, and the DNA alignment with E. coli K12. a) gene pepN, b) gene ydaN, and c) gene gdhA. (0.96 MB PDF) [file pone.0007526.s002.pdf]
