# Supplementary material for: Genome-Wide Identification of Transcription Start Sites, Promoters and Transcription Factor Binding Sites in E. coli
Source: PLoS One. 2009 Oct 19;4(10):e7526. doi: 10.1371/journal.pone.0007526 (PMC2760140; doi:10.1371/journal.pone.0007526)
Supplement: Table S5 — Transcriptional factors binding sites associated to each TSS. The information in RegulonDB for each of these TFs and their binding sites is shown. (0.05 MB DOC) [file pone.0007526.s011.doc]

Table S5

| **TF Name** | **Regulated genes in RegulonDB** | **Binding Sites in RegulonDB** | **TF Name** | **Regulated genes in RegulonDB** | **(Binding Sites in RegulonDB** |
| --- | --- | --- | --- | --- | --- |
| AraC | 5 | 13 | MelR | 2 | 5 |
| ArcA | 59 | 85 | MetJ | 9 | 24 |
| ArgR | 15 | 26 | MetR | 6 | 5 |
| CRP | 182 | 213 | Nac | 8 | 13 |
| CsgD | 4 | 5 | NarL | 27 | 81 |
| CysB | 9 | 10 | NarP | 10 | 19 |
| CytR | 9 | 11 | OmpR | 12 | 23 |
| DeoR | 3 | 8 | OxyR | 12 | 12 |
| FNR | 98 | 77 | PhoB | 14 | 21 |
| FadR | 9 | 12 | PhoP | 20 | 19 |
| Fis | 90 | 213 | PurR | 19 | 20 |
| FlhDC | 20 | 19 | RcsAB | 9 | 11 |
| FruR | 21 | 13 | RhaS | 2 | 4 |
| Fur | 40 | 66 | Rob | 11 | 7 |
| GlpR | 4 | 18 | SoxS | 18 | 19 |
| GntR | 6 | 10 | TorR | 5 | 7 |
| HipB | 1 | 4 | TrpR | 5 | 10 |
| IHF | 75 | 94 | TyrR | 9 | 19 |
| IclR | 2 | 10 | XylR | 2 | 4 |
| IscR | 7 | 10 |  |  |  |
| LexA | 24 | 29 |  |  |  |
| Lrp | 33 | 54 |  |  |  |
| MalT | 5 | 15 |  |  |  |
